# A Review on Pre-, In-Process, and Post-Synthetic Strategies to Break the Surface Area Barrier in g-C_3_N_4_ for Energy Conversion and Environmental Remediation

**DOI:** 10.3390/nano15130956

**Published:** 2025-06-20

**Authors:** Mingming Gao, Minghao Zhao, Qianqian Yang, Lan Bao, Liwei Chen, Wei Liu, Jing Feng

**Affiliations:** 1 College of Biological and Chemical Engineering, Qilu Institute of Technology, Jinan 250200, China; 2 Key Laboratory of Superlight Materials & Surface Technology of Ministry of Education, Harbin Engineering University, Harbin 150001, China

**Keywords:** specific surface area, polymeric carbon nitride, controllable synthesis, top-down approaches, bottom-up strategies

## Abstract

Nanomaterials with large specific surface area (SSA) have emerged as pivotal platforms for energy storage and environmental remediation, primarily due to their enhanced active site exposure, improved mass transport capabilities, and superior interfacial reactivity. Among them, polymeric carbon nitride (g-C_3_N_4_) has garnered significant attention in energy and environmental applications owing to its visible-light-responsive bandgap (~2.7 eV), exceptional thermal/chemical stability, and earth-abundant composition. However, the practical performance of g-C_3_N_4_ is fundamentally constrained by intrinsic limitations, including its inherently low SSA (<20 m^2^/g via conventional thermal polymerization), rapid recombination of photogenerated carriers, and inefficient charge transfer kinetics. Notably, the theoretical SSA of g-C_3_N_4_ reaches 2500 m^2^/g, yet achieving this value remains challenging due to strong interlayer van der Waals interactions and structural collapse during synthesis. Recent advances demonstrate that state-of-the-art strategies can elevate its SSA to 50–200 m^2^/g. To break this surface area barrier, advanced strategies achieve SSA enhancement through three primary pathways: pre-treatment (molecular and supramolecular precursor design), in process (templating and controlled polycondensation), and post-processing (chemical exfoliation and defect engineering). This review systematically examines controllable synthesis methodologies for high-SSA g-C_3_N_4_, analyzing how SSA amplification intrinsically modulates band structures, extends carrier lifetimes, and boosts catalytic efficiencies. Future research should prioritize synergistic multi-stage engineering to approach the theoretical SSA limit (2500 m^2^/g) while preserving robust optoelectronic properties.

## 1. Introduction

With the rapid development of global industrialization, energy shortages and environmental pollution have emerged as critical challenges worldwide. Addressing these issues requires dual approaches, including exploring alternative clean energy sources and developing advanced technologies for environmental remediation. In this context, semiconductor materials with tailored photoelectrochemical properties have attracted significant attention, owing to their ability to harness solar energy for sustainable applications [1,2,3,4]. Notably, nanostructured semiconductors with large specific surface areas (SSA) have been extensively investigated, as their enhanced surface reactivity, abundant active sites, and improved mass transport properties can substantially boost catalytic performance. Representative materials like activated carbon (with SSA up to 3000 m^2^/g) and graphene (featuring a 2D layered structure) exemplify how a high SSA enables exceptional adsorption capacity and electron transfer efficiency [5,6,7,8,9]. However, unlike conductive materials, semiconductors require precisely engineered band structures to balance light absorption and redox potential. Integrating the unique advantages of semiconductors with high SSA therefore represents a promising strategy to optimize energy use and tackle environmental crises.

Among semiconductor candidates, polymeric carbon nitride (g-C_3_N_4_), composed of earth-abundant carbon and nitrogen elements, has stood out as a metal-free photocatalyst since its discovery for water splitting in 2009 [10]. Its appropriate bandgap (~2.7 eV), visible-light responsiveness, and exceptional thermal/chemical stability (derived from triazine or 3-s-triazine ring units) have enabled widespread applications in photocatalytic hydrogen evolution, CO_2_ reduction, organic pollutant degradation, and so on [11,12,13,14,15,16]. Nevertheless, the practical performance of bulk g-C_3_N_4_ remains far below theoretical expectations due to inherent limitations [17] including low SSA (<20 m^2^/g) caused by strong interlayer van der Waals interactions and incomplete exfoliation during conventional thermal polymerization, rapid recombination of photogenerated carriers due to insufficient active sites and inefficient charge separation, and l.imited depth of light absorption resulting from particle aggregation. Intriguingly, the theoretical SSA of g-C_3_N_4_ nanosheet (up to 2500 m^2^/g) suggests immense potential for improvement through nanostructure engineering and surface modification, thereby addressing these bottlenecks [18].

Significant efforts have been devoted to enhancing the SSA of g-C_3_N_4_ via morphological control (e.g., porous architectures, nanotubes, nanosheets) and defect engineering [19,20,21,22]. These strategies not only increase the exposure of active sites but also modulate electronic structures to improve light absorption and charge transfer kinetics. For instance, 2D g-C_3_N_4_ nanosheets exhibit enhanced photocatalytic activity due to their ultrathin structure and shortened carrier migration paths [23,24]. However, critical trade-offs persist. Quantum size effects in nanostructured g-C_3_N_4_ may inadvertently widen the bandgap, compromising visible-light utilization, and excessive defects (e.g., pores, vacancies) can act as recombination centers, counteracting SSA benefits. Interplay between morphology, band structure, and defect density requires delicate balancing to optimize overall performance.

While prior reviews have comprehensively cataloged general modification strategies for g-C_3_N_4_, they have seldomed examined surface area engineering through the critical lens of process chronology. Specifically, the distinct roles of pre-treatment (precursor design), in-process (polymerization control), and post-processing (exfoliation/functionalization) strategies remain underexplored in correlating SSA enhancement with application-specific performance. This review bridges that gap by establishing a unified framework, systematically evaluating how interventions at each phase uniquely govern pore architecture and active site density to break the surface area barrier, enabling optimal energy use and environmental applications.

**Figure 1:**
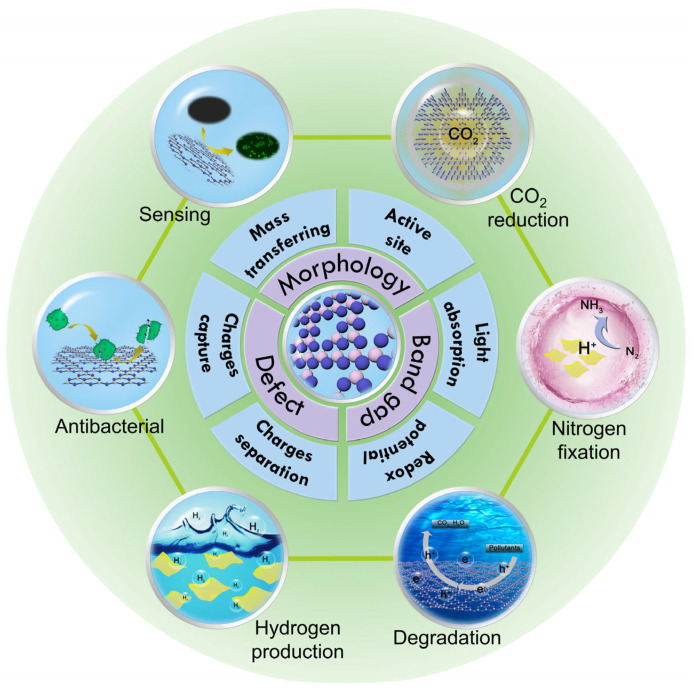
Schematic illustration of high-SSA g-C_3_N_4_ for various applications.

This review rigorously examines advances in high-SSA g-C_3_N_4_ synthesis through three chronologically defined pathways, focusing on pre-treatment strategies (precursor molecular engineering and supramolecular assembly), in-process modulation (templating and salts prior to polymerization), post-processing techniques (Chemical/thermal exfoliation, defect etching, and ultrasonication), structure–property relationships (how SSA enhancement influences band structure, carrier dynamics, and catalytic efficiency), and multifunctional applications (environmental remediation, energy conversion, and emerging biomedical uses (Figure 1)). Finally, we critically analyze remaining challenges (e.g., scalability, stability) and propose future research directions to guide the rational design of high-performance g-C_3_N_4_-based materials with optimized surface architectures.

## 2. The Method of Synthesizing High-SSA Carbon Nitride

The aforementioned analysis of application trends reveals that the performance of g-C_3_N_4_ in energy conversion and environmental remediation is critically dependent on its surface/interface properties. However, bulk g-C_3_N_4_ prepared by conventional methods suffers from low specific surface area (<20 m^2^/g) and insufficient active site exposure, which fundamentally limit its photocatalytic/electrocatalytic efficiency. To address these bottlenecks, recent research has focused on controlled synthesis strategies at the molecular level, including topological structure design, defect engineering, and heterojunction construction, to precisely tailor the specific surface area and surface chemistry of g-C_3_N_4_. The following section systematically explores synthetic paradigms for high SSA g-C_3_N_4_, elucidating how these strategies optimize mass transport pathways, enhance light harvesting, and promote charge carrier separation, thereby providing a material foundation to overcome the application challenges discussed above.

### 2.1. Structure of Carbon Nitride

**Figure 2:**
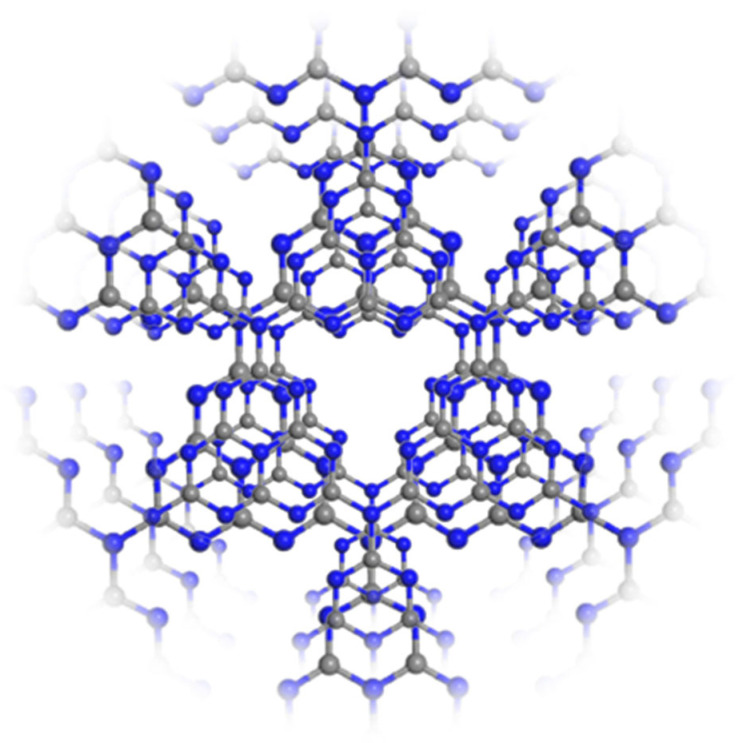
Layer stacking structure and basic units of g-C_3_N_4_.

In the structure of g-C_3_N_4_, the C and N atoms are both *sp2* hybridized and their *p* orbitals have overlapped to form delocalized Π-bond. The basic units are connected to each other by N atoms at the end to form a planar grid structure; g-C_3_N_4_ then presents a layer stacking structure, which is similar to that of graphite (Figure 2). The DFT study of g-C_3_N_4_ illustrates that it has the special properties of a semiconductor, such as a suitable band gap (~2.6 eV), and the appropriate positions of valence band and conduction band. These results indicate that g-C_3_N_4_ has a certain redox capacity in theory (oxidizing water to produce oxygen, reducing water to produce hydrogen), which lays the foundation for its application in the field of photocatalytic hydrogen production.

### 2.2. Preparation of Carbon Nitride

g-C_3_N_4_ is usually formed by a precursor that contains C and N elements, through thermal (550–600 °C) polymerization, which represents an attractive method due to its simplicity and low cost. Cyanamide, dicyandiamide, melamine, urea, and thiourea commonly serve as N-rich precursors. In the case of melamine as the precursor, a 3-s-triazine ring can be formed after the polymerization and rearrangement of melamine. Condensation into g-C_3_N_4_ then continues with the 3-s-triazine ring as the basic unit. NH_3_ is released during the polymerization process. Therefore, the structural properties of g-C_3_N_4_ strongly depend on the synthesis conditions.

### 2.3. Preparation of High-SSA Carbon Nitride

In addition, researchers frequently employ templating methods, precursor selection and processing, pyrolysis conditions, among others, to prepare g-C_3_N_4_ with large SSA. In recent years, solvothermal methods and supramolecular assembly techniques have also gained prominence. Here, we present a statistical overview of these various approaches, categorizing them comprehensively, and detailing the intricacies, advantages, and limitations of each method. The preparation methods of g-C_3_N_4_ with large SSA mainly focus on the following three aspects. High-SSA g-C_3_N_4_ with a special structure has been prepared by changing the thermal polymerization mode of the N-rich precursors through special pretreatment. Processing during the synthesis of g-C_3_N_4_ by introducing a template and salt reagent can prepare g-C_3_N_4_ with a special morphology. Post-processing of the synthesized g-C_3_N_4_ by adding energy and acid/base reagents can be used to destroy the interlayer forces and produce pore structures. This critical review presents a hierarchical analysis framework for synthesizing high-SSA g-C_3_N_4_ materials through a tripartite approach: precursor engineering (pre-treatment), in-process treatment, and post-synthesis. The established structure–property correlation matrix enables rational selection of synthesis protocols based on application-specific performance metrics.

#### 2.3.1. Pre-Treatment of Carbon Nitride

##### The Influence of Raw Materials on the Specific Surface Area and Morphology

**Figure 3:**
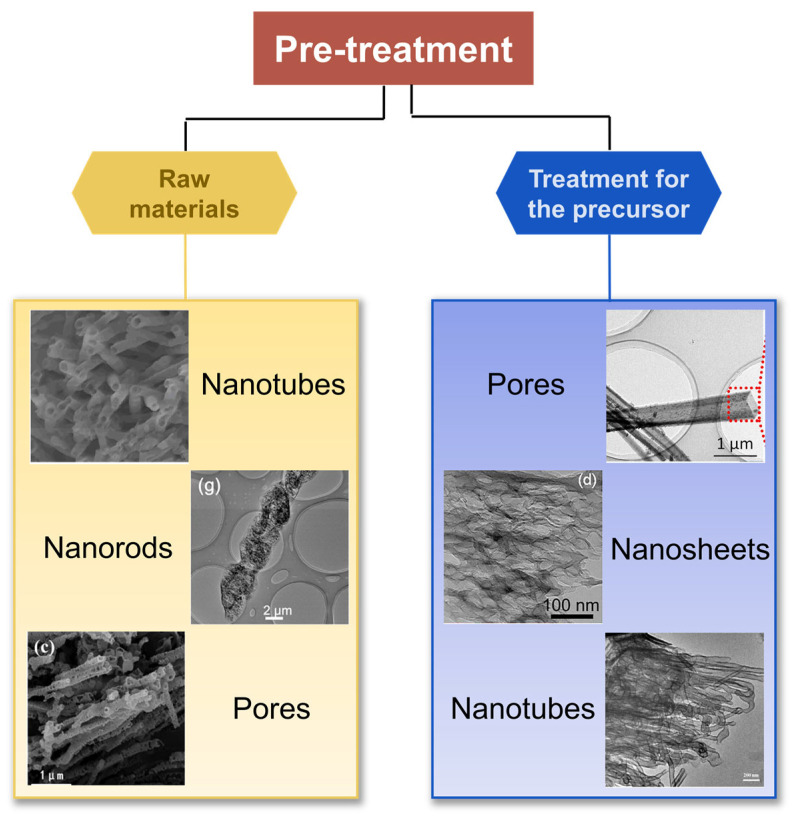
Pre-treatment of high-SSA g-C_3_N_4_ [20,25,28,36,38,43].

**Figure 4:**
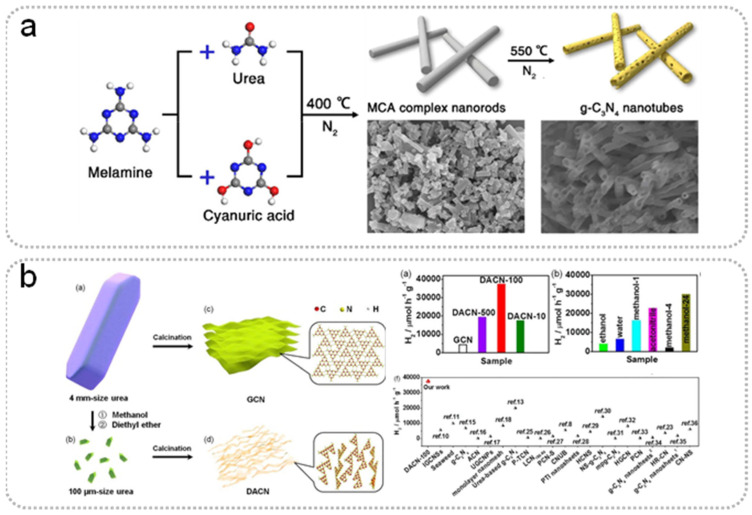
(**a**) The formation process of hollow C_3_N_4_ nanotubes. Reproduced with permission [28]. Copyright © 2019 Springer Nature. (**b**) Preparation process and photocatalytic activity of defect-rich amorphous carbon nitride; hydrogen-evolution rate for defect-rich amorphous carbon nitride in comparison with other GCN-based photocatalysts. Reproduced with permission [36]. Copyright © 2018 American Chemical Society.

The selection and mix of raw materials are part of the simple, template-less, one-step polymerization method for the preparation of g-C_3_N_4_ with high SSA (Table 1) [20,21,25,26,27,28,29]. When urea is used as a precursor, CO_2_ is released simultaneously, resulting in the synthesized g-C_3_N_4_ showing a more porous structure and larger SSA. Additionally, it is worth noting that the mixed raw materials can be assembled into a supramolecular precursor by hydrogen bonding in solution [30]. The structure of the prepared g-C_3_N_4_ ultimately depends on the supramolecular precursor, because the melting point of supramolecular precursor is higher than the formation temperature of bulk g-C_3_N_4_ during thermal polymerization (Figure 3). Wang et al. prepared g-C_3_N_4_ nanotubes through thermal polymerization by mixing urea with melamine (mass ratio = 10:1) [28]. In thermal polymerization, urea can be converted to cyanuric acid at 400 °C, which then combined with melamine by hydrogen bonding to form supramolecular nanorods. Therefore, the formation of supramolecular precursors is closely related to the mass ratio of urea and melamine and further guides the formation of nanotubes (Figure 4a). Chen et al. prepared 3D g-C_3_N_4_ with a SSA of 130 m^2^ g^−1^ by mixing melamine and cyanic acid (at a molar ratio of 1:1) [29]. The 3D interconnected open-framework provided a carrier transport channel for highly efficient photocatalytic overall water splitting (splitting pure water into H_2_ and O_2_ with high evolution rate up to 101.4 and 49.1 µmol g^−1^ h^−1^). Bao et al. designed a simple template-mediated approach by supramolecular self-assembly (Cu-melamine-cyanuric acid) to prepare a copper-doped porous g-C_3_N_4_ (Cu-pCN) photocatalyst, increasing the SSA from 11.37 to 142.8 m^2^ g^−1^ [31].

##### The Treatment for the Precursor of Carbon Nitride

The synthesis of g-C_3_N_4_ with special nanostructure is undoubtedly one of the effective ways to improve its specific surface area. As the performance of g-C_3_N_4_ depends on its nitrogen precursor, some treatment methods for the precursor have gradually attracted wide attention. Apart from the precursor mixing, treatment for the precursor of g-C_3_N_4_ has attracted increasing attention because the properties of g-C_3_N_4_ are closely associated with the nitrogen-rich precursor (Figure 3). This method means that the precursor is treated with acid [32,33,34,35], organic solvent [36,37], and hydrothermal process [38,39,40,41] before the normal calcination process. Through pretreatment, the precursor can form a more abundantly porous structure during the subsequent thermal polymerization or solvothermal reaction, thereby significantly increasing the specific surface area of the g-C_3_N_4_. The enlargement of the specific surface area implies more active sites for catalytic reaction, which contributes to enhancing the catalytic performance of g-C_3_N_4_. Additionally, pretreatment can also assist in controlling the morphology of g-C_3_N_4_, such as achieving a sheet-like or porous plate-like structure. These specific morphologies facilitate the migration of photogenerated carriers and the mass transfer of reactants, further enhancing the material’s photocatalytic performance.

Dong et al. prepared g-C_3_N_4_ with a porous ultrathin nanosheet structure by calcining HCl-treated melamine [32]. The thermal polymerization mode of HCl-treated melamine is different from the traditional mode, due to the HCl reacting with the amino groups of melamine. The production of voids increased the specific surface area (345 m^2^ g^−1^) and reduced the charge migration distance (from body to surface), resulting in an excellent NO photocatalytic removal rate. Zhang et al. speculated that acid could change the growth orientation of the precursor, which would be beneficial to the increase of active sites [33]. Additionally, a defect-rich amorphous g-C_3_N_4_ photocatalyst (228.4 m^2^ g^−1^) was synthesized by simple direct calcination of the rationally size-reduced urea crystals, which was effectively controlled by the anti-solvent growth method [36] (Figure 4b). The introduction of N vacancies caused a broad visible-light-responsive range, exposed surface bonding sites, and quenched radiative recombination, leading to enhanced photocatalytic activity for hydrogen production (37,680 µmol g^−1^ h^−1^ under visible-light irradiation). Additionally, hydrothermal processes have also been used to change the precursor, affecting the structure of g-C_3_N_4_. The hydrolytic product of the precursor can assemble with the precursor to form a supramolecular precursor. Mo et al. synthesized g-C_3_N_4_ nanotubes with a specific surface area of 127.8 m^2^ g^−1^ by calcining the hydrothermal treated melamine [38]. Compared with bulk g-C_3_N_4_, the g-C_3_N_4_ nanotubes exhibited an excellent hydrogen evolution rate (118.5 µmol h^−1^), which was ascribed to the nanotube structure and the N defects. Moreover, Cheng et al. suggested that the hydrolytic products of dicyandiamide (amidine urea) can weaken the interaction between Π–Π stacking, which is helpful for generating g-C_3_N_4_ in thin layers [40]. Therefore, the precursor pretreatment can not only change the morphology but also optimize the electronic structure of g-C_3_N_4_. However, the performances of the obtained g-C_3_N_4_ are closely related to the details of the treatment, such as the concentration of acid, organic solvent, and the temperature and duration of the hydrothermal process (Table 2) [32,33,34,35,36,37,38,39,40,42,43]. In order to achieve high-performance g-C_3_N_4_, the optimization of details in the pretreatment process needs to be further considered.

#### 2.3.2. In-Process Preparation of Carbon Nitride

##### Introducing the Template During the Synthesis

Template methods are widely used for precise control of the morphology and porous structure of materials, according to a variety of templates. The prepared g-C_3_N_4_ by template method usually displays a mesoporous structure and a large specific surface area. Common templates for preparing g-C_3_N_4_ include SBA-15, MCM-22, SiO_2_, KCC-1 silica spheres (Table 3) [44,45,46,47,48,49,50,51,52,53,54,55,56,57,58]. SBA-15 has been used as a template to synthesize g-C_3_N_4_ with uniform mesoporous structure, showing a high specific surface area of 505 m^2^ g^−1^ [44]. Cui et al. prepared g-C_3_N_4_ with specific surface areas of 188 m^2^ g^−1^ and 239 m^2^ g^−1^ using SiO_2_ and SBA-15, respectively [47]. This shows that the morphologies and specific surface areas of the prepared g-C_3_N_4_ depend on the template used. In addition, Vinu et al. synthesized mesoporous g-C_3_N_4_ (239 m^2^ g^−1^) with different pore sizes, using SBA-15 [45]. It should be noted that the pore volume, pore size, specific surface area, and N content of the prepared g-C_3_N_4_ can be controlled by the simple adjustment of the ethylenediamine–carbon tetrachloride weight ratio. Therefore, the structural properties of the prepared g-C_3_N_4_ are also related to the precursor; the well mixing between precursor and template is one of the important steps of the template method. For example, Zhao et al. prepared ordered mesoporous g-C_3_N_4_ nanorods by using hexamethylenetetramine as the precursor, which had high specific surface area (1116 m^2^ g^−1^), a bimodal mesoporous structure, and high N content [48]. Kailasam et al. mixed cyanamide (precursor for g-C_3_N_4_) directly with tetraethyl silicate (precursor for silica) using the sol–gel method to ensure the simultaneous production of g-C_3_N_4_ and SiO_2_ and achieved full mixing [51].

**Figure 5:**
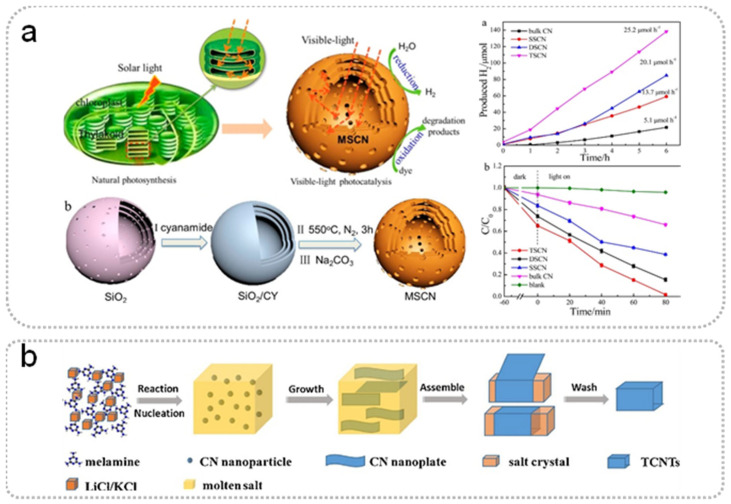
(**a**) Schematic illustration of the preparation, photocatalytic H_2_ production, and photocatalytic degradation of MSCN nanocapsules. Reproduced with permission [53]. Copyright © 2016 American Chemical Society. (**b**) Schematic illustration of the formation process of tetragonal carbon nitride hollow tubes in the molten salt medium. Reproduced with permission [43]. Copyright © 2018 Elsevier.

Besides that, the g-C_3_N_4_ with special nanostructures have been prepared by making various special templates. Inspired by natural photosynthesis, Tong et al. prepared the multiple-shell g-C_3_N_4_ (310.7 m^2^ g^−1^) with excellent photocatalytic hydrogen production activity, imitating the excellent light capture ability and water storage structure of chloroplasts (Figure 5a) [53]. The successful preparation of this structure depended on the self-made three-layer spherical SiO_2_ template with a porous structure. Specifically, the existence of pores in the SiO_2_ template ensured the full mixing between cyanamide and template. Lin et al. synthesized mesoporous g-C_3_N_4_ (132.26 m^2^ g^−1^) with a chiral supramolecular helical structure and liquid crystal layered structure via prepared chiral mesoporous SiO_2_, exhibiting an ultrahigh hydrogen evolution rate of 219.9 µmol h^−1^ under visible-light irradiation compared with bulk g-C_3_N_4_ [54]. Liu et al. mixed mesoporous SiO_2_ nanorods and cyanamide several times to prepare mesoporous g-C_3_N_4_ nanotubes with a specific surface area of 135.1 m^2^ g^−1^ using a nano-confined reaction inside silica nanotubes with porous shells [55]. Moreover, dicyandiamide and melamine can also be used in the preparation of g-C_3_N_4_ with a high specific surface area by changing the mixing mode of the template and precursor. For example, onion-like ring g-C_3_N_4_ was prepared using melamine and SiO_2_ [56]. The vapor deposition method was selected for the synthesis, because solid melamine is easy to sublimate in the pyrolysis process. Si et al. synthesized hierarchical porous PCN microspheres (Hydrogen evolution activity of 4635.8 µmol h^−1^ g^−1^) via in situ precursor conversion technology utilizing the solubility difference between cyanamide and dicyandiamide in aqueous solution [57]. After the addition of NH_4_OH, the precursor started to transform from cyanamide to dicyandiamide along the wall surfaces of the hierarchical porous SiO_2_ microspheres. This procedure can overcome the low solubility of dicyandiamide and avoid the evaporation of cyanamide.

##### Introducing the Salt During the Synthesis

Moreover, the polymerization process of the precursor can be affected by salt [15,42,43,59]. Salts with low melting points gradually melt to form a liquid phase under high-temperature conditions. The raw materials undergo reactions in the liquid phase of the molten salt, which not only facilitates the mass transfer and diffusion processes of the reactant particles but also benefits crystal growth. Additionally, some molten salt particles penetrate into the gaps between the particles of the generated product, preventing the aggregation of product particles and improving the crystal structure of the sintered product. For example, Tian et al. controlled the synthesis of g-C_3_N_4_ hollow tubes with a specific surface area of 128 m^2^ g^−1^ through the molten salt method [43]. First, the melamine was oversaturated and nucleated in advance by adding a LiCl/KCl eutectic mixture during the polymerization process. Then, the formed nanoparticles combined and grew into nanosheets in the presence of highly soluble Li^+^, K^+^, and Cl^−^. Finally, the nanosheets curled to form the g-C_3_N_4_ hollow tube due to the minimization of surface free energy (Figure 5b). In addition, N species could easily be captured in the living salt medium, leading to a high N content in the synthesized g-C_3_N_4_.

#### 2.3.3. Post-Processing of Carbon Nitride

**Figure 6:**
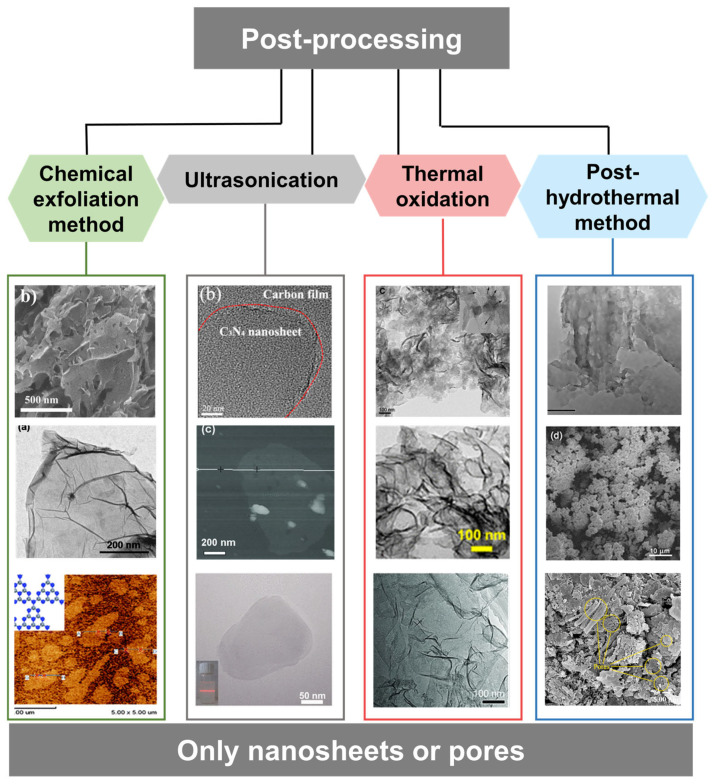
Pre-treatment of high-SSA g-C_3_N_4_ [60,63,65,68,69,70,71,72,73,74,75,76].

Similar to the reason why it is difficult to exfoliate layers of graphene, the layers of g-C_3_N_4_ agglomerate due to the presence of van der Waals forces, resulting in a relatively low specific surface area. Inspired by the Hummers method of preparing graphene, a series of post-processing methods for exfoliating g-C_3_N_4_ have been developed due to its graphite-like layered stacking structure. g-C_3_N_4_ can be intercalated by acids and organic solvents. The interlayer forces are destroyed by the introduced energy (ultrasonic, thermal energy, high temperature, and pressure) to form g-C_3_N_4_ nanosheets. In addition, the addition of base can cause reactions with C or N to form porous structures. All the above approaches can increase the specific surface area of g-C_3_N_4_ (Figure 6). Recently, each method has been combined with other methods to exfoliate g-C_3_N_4_ in a more convenient, energy-saving, and efficient was. The large surface area of the g-C_3_N_4_ nanosheets that are produced can increase the area of contact with reactants. Moreover, the nanosheets’ structure shortens the migration path of photo-generated carriers from the catalyst’s bulk to its surface, which effectively inhibits the recombination of photo-generated carriers during the transfer process.

##### Acid/Chemical Treatment

In an acidic solution, hydrogen ions (H^+^) undergo a protonation reaction with the nitrogen atoms on the surface of g-C_3_N_4_ or other basic sites, significantly increasing the electrostatic repulsion between the layers, effectively weakening the van der Waals forces between the layers and reducing the bonding strength between them. Meanwhile, the treatment of g-C_3_N_4_ in liquid acid is a means of non-oxidized intercalation, exfoliation, and surface modification, which can preserve the C-N heterocyclic skeleton and avoid the excessive production of defects [14,60,61]. Numerous works have combined acid with ultrasonication or thermal treatment to increase the specific surface area of g-C_3_N_4_ efficiently [62,63,64,65].

**Figure 7:**
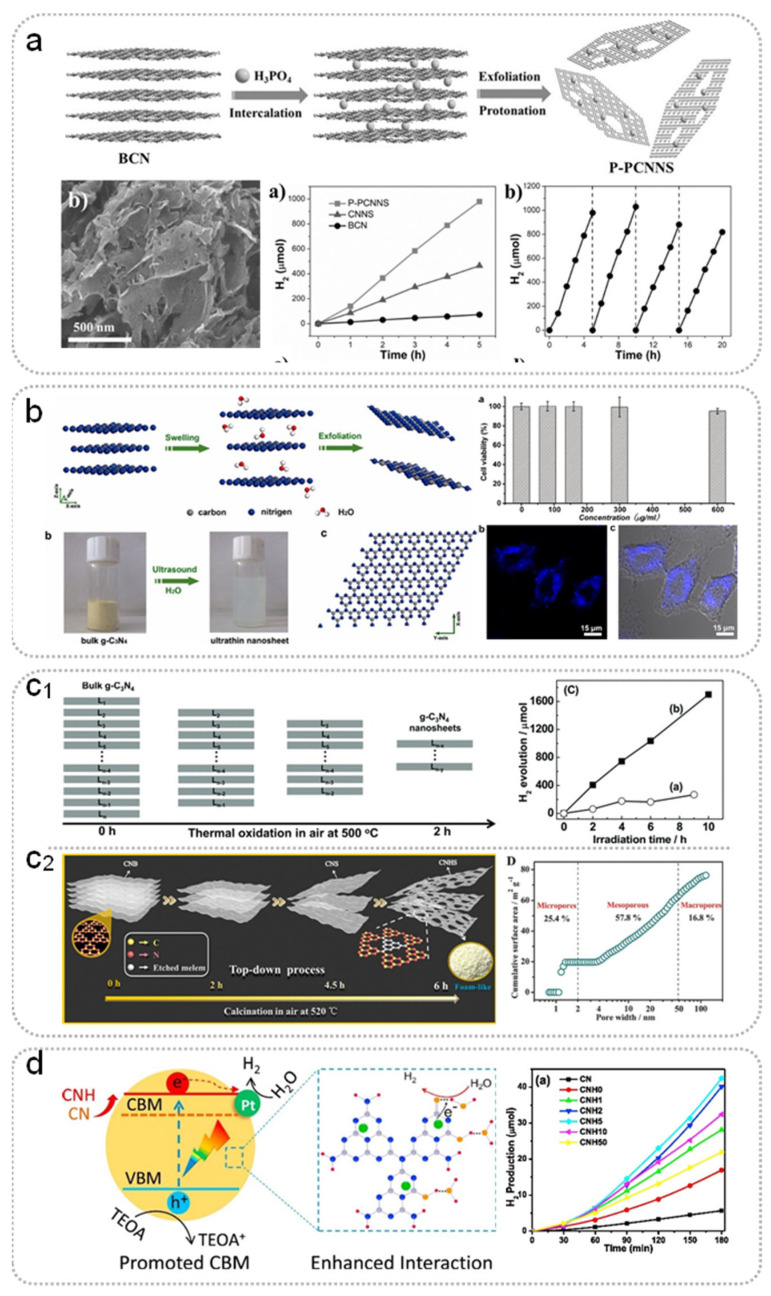
(**a**) Illustration of the preparation process, SEM images, photocatalytic activity for H_2_ evolution, and photocatalytic H_2_ evolution stability of P-PCNNS under visible light. Reproduced with permission [60]. Copyright © 2016 WILEY-VCH. (**b**) Schematic illustration of liquid-exfoliation process, suspension, and crystal structure of ultrathin C_3_N_4_ nanosheets, viability after 48 h, confocal fluorescence image, and overlaid bright-field image of HeLa cells incubated with ultrathin C_3_N_4_ nanosheets. Reproduced with permission [68]. Copyright © 2012 American Chemical Society. (**c_1_**) Schematic of the formation process of C_3_N_4_ nanosheets and hydrogen evolution from water under UV-visible light. Reproduced with permission [69]. Copyright © 2012 WILEY-VCH. (**c_2_**) Top-down process for preparation and cumulative surface area of foam-like hollow ultrathin C_3_N_4_ nanosheets. Reproduced with permission [77]. Copyright © 2016 WILEY-VCH. (**d**) The intralayer chemical structure of C_3_N_4_ and the amount of H_2_ versus time after hydrothermal treatment. Reproduced with permission [70]. Copyright © 2018 Elsevier.

For example, g-C_3_N_4_ ultra-thin two-dimensional nanosheets with a micro-mesoporous structure were prepared by inserting H_3_PO_4_ into g-C_3_N_4_ layers [60]. The obtained g-C_3_N_4_ showed a larger specific surface area (increasing from 7.6 m^2^ g^−1^ to 55.4 m^2^ g^−1^) and more transverse charge transfer channel to promote the separation of the photo-generated carriers (Figure 7a). The average H_2_ evolution rate under visible light is 195.8 µmol h^−1^, which is much higher than that of bulk g-C_3_N_4_ (14.5 µmol h^−1^). Du et al. synthesized large-scale soluble acidified g-C_3_N_4_ (42 m^2^ g^−1^) via H_2_SO_4_ acidification, expanding the functionalization and application of g-C_3_N_4_ [61]. Bulk g-C_3_N_4_ was exfoliated using a combination of H_2_SO_4_ and ultrasonication to form nanosheets (25.7 m^2^ g^−1^); with a higher reaction rate of p-nitrophenol photo-reduction due to the positively charged surface and the nanosheets’ morphology [62]. In the same way, monolayer C_3_N_4_ nanosheets with a specific surface area of 205.8 m^2^ g^−1^ were obtained through sonicated exfoliation after H_2_SO_4_ treatment, showing a threefold enhancement in photocatalytic H_2_ production [65]. Ma et al. selected HCl in combination with sonication; the as-prepared ultrathin g-C_3_N_4_ nanosheets had a high specific surface area of 305 m^2^ g^−1^ and reached the lowest heparin detection limit of 18 ng mL^−1^ [63]. Thus, it can be seen that the exfoliation effect on g-C_3_N_4_ is closely related to the type and concentration of the liquid acid, the mixing time, and the follow-up details (such as speed at subsequent centrifugation). The application of this method is restricted by uneven treatment, multiple influencing factors, long processing time, and low yield. In addition, g-C_3_N_4_ nanosheets prepared via liquid acid exfoliation showed a wider band gap due to the quantum size effect, resulting in diminished visible-light response (Table 4) [60,61,62,63,64,65,66,67].

In addition, the Hummers method had been used to exfoliate bulk g-C_3_N_4_, due to its graphite-like structure [66,67]. Teng et al. synthesized ultrathin g-C_3_N_4_ nanosheets using combination of intercalation with H_2_SO_4_, oxidant-assisted exfoliation of KMnO_4_, and reduction of HHA [66]. The obtained g-C_3_N_4_ had a large specific surface area of 355.8 m^2^ g^−1^, a wider band gap of 3.07 eV, and an increased lifetime. However, the structure of g-C_3_N_4_ may be destroyed by the use of strong oxidants, resulting in collapse of the skeleton or an increase in defects.

**Table 4:** Influencing factors, assistant methods, and results of adding acids/chemical exfoliation [60,61,62,63,64,65,66,67].

References	Synthesis of Bulk g-C_3_N_4_	Acid				Assistant Method	Results			
		M_bulk g-C3N4_	Type	Concentration or Volume	Treated Time		Band Gap	BET	Morphology	Application
[60]	Dicyandiamide 550 °C for 4 h at 5 °C min^−1^	0.2 g	H_3_PO_4_	0.3 mL	3 d	Dispersed in 20 mL dimethylformamide	2.63 eV ↓ 2.74 eV	7.6 m^2^/g ↓ 55.4 m^2^/g	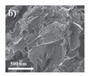	Photocatalytic hydrogen evolution and CO_2_ conversion
[61]	Dicyandiamide 550 °C for 4 h at 2.3 °C min^−1^	4 g	H_2_SO_4_ (98%)	52 g	5 h	20 g oleum NH_4_Cl (1.60 mol)	2.83 eV ↓ 3.78 eV	12 m^2^/g ↓ 42 m^2^/g	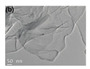	-
[62]	Melamine	2 g	H_2_SO_4_	40 mL 98 wt%	10 h	Sonicated with 8 h	2.64 eV ↓ 2.68 eV	8.1 m^2^/g ↓ 25.7 m^2^/g	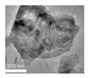	Photo-reduction of p-nitrophenol
[63]	Dicyandiamide 550 °C for 4 h at 2.3 °C min^−1^ in static air	1 g	HCl	25 mL 10 M	1 h	Sonication for 2 h	2.57 eV ↓ 2.75 eV	9 m^2^/g ↓ 305 m^2^/g	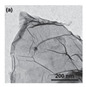	Excellent metal-/label-free biosensing platform
[64]	Melamine 550 °C for 4 h at 3 °C min^−1^ in static air	1.0 g	H_2_SO_4_	10 mL 98 wt%	8 h	Calcinated at 550 °C in N_2_ for 2 h	2.65 eV ↓ 2.79 eV	12 m^2^/g ↓ 54.3 m^2^/g	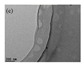	Photocatalytic degradation
[65]	Dicyandiamide 550 °C for 4 h at 2.3 °C min^−1^ in static air	1 g	H_2_SO_4_	10 mL 98 wt%	8 h	Sonication	2.64 eV ↓ 2.92 eV	4.3 m^2^/g ↓ 205.8 m^2^/g	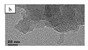	Photocatalytic hydrogen evolution
[66]	-	1 g	H_2_SO_4_	30 mL 98 wt%	-	Hummer’s method	2.54 eV ↓ 3.07 eV	3.5 m^2^/g ↓ 355.8 m^2^/g	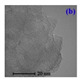	-
[67]	Dicyandiamide 500 °C for 1 h 520 °C for 3 h	10 g	H_2_SO_4_	230 mL 98 wt%	-	Hummer’s method	2.72 eV ↓ 3.85 eV	-	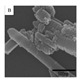	Photocatalytic degradation

##### Ultrasonication

Liquid-phase ultrasound has attracted much attention due to its simple and mild exfoliation process, which can exfoliate bulk g-C_3_N_4_ to achieve a nanosheet structure [68,71,72,78]. The matching degree of the surface energy between the used solvent and g-C_3_N_4_ determines effective exfoliation via liquid-phase ultrasound. The relationship between the surface energy and mixing enthalpy can be described by the following empirical formula [71]:

(1) $$\frac{\Delta H_{mix}}{V_{mix}}=\frac{2}{T_{bulk}}{\left(\delta_{G}-\delta_{sol}\right)}^{2}\phi$$

where Δ*H* is the enthalpy of mixing, *δ* is the square root of the component surface energy, *T_bulk_* is the average thickness of g-C_3_N_4_, and *ϕ* is the volume fraction of g-C_3_N_4_. It is well known that the bulk g-C_3_N_4_ can be effectively exfoliated by liquid-phase ultrasound when the mixing enthalpy is minimized; that is, when the surface energy of g-C_3_N_4_ and the solvent match.

Liquid-phase ultrasound is usually selected in combination with other methods to exfoliate bulk g-C_3_N_4_ in order to increase its specific surface area. For example, ultrathin g-C_3_N_4_ nanosheets have been successfully prepared in water via green liquid phase exfoliation, and it has been used in biological imaging (Figure 7b) [68]. In detail, the bulk g-C_3_N_4_ was treated ultrasonically in water (16 h) to obtain atom-thick nanosheets. The prepared g-C_3_N_4_ nanosheets showed enhanced capacity for light absorption and response, inducing an extremely high PL quantum yield, beneficial for bioimaging application. Meanwhile, organic solvents have also been selected as dispersion media for liquid-phase ultrasound due to their own functional groups [71,72,78]. Yang et al. synthesized g-C_3_N_4_ nanosheets by liquid-phase ultrasound in IPA [71], with a high specific surface area (384 m^2^ g^−1^) and a large aspect ratio, beneficial for photocatalytic hydrogen evolution. She et al. compared the exfoliation effect between butanediol and 1.3-BUT on bulk g-C_3_N_4_, demonstrating the influence of functional groups on the exfoliation process [78]. The existence of two hydroxyl groups (1.3-BUT) leads to more effective exfoliation of g-C_3_N_4_, resulting in the enhanced microelement-sensing capability. Moreover, Lin et al. conducted ultrasonic treatment of g-C_3_N_4_ with mixed solvent as the dispersing medium [72]. In the aqueous solution with adjustable concentration, g-C_3_N_4_ nanosheets (59.4 m^2^ g^−1^) were prepared by changing the volume ratio of the two solvents. Thus, the properties of the organic solvent are closely related to the exfoliation effect of the liquid-phase ultrasound.

In addition, the interlayer forces and surface properties of g-C_3_N_4_ can be influenced by its crystallization and polymerization. Thus, the exfoliation effect of liquid-phase ultrasonic treatment is also related to the method of synthesis of the g-C_3_N_4_. The application of the liquid-phase ultrasonic method has been limited due to its long processing time and unsatisfactory yield (Table 5) [68,71,72,78]. Recently, liquid-phase ultrasonic has gradually become an auxiliary method to prepare high-surface-area g-C_3_N_4_.

##### Thermal Oxidation Treatment

It is well known that g-C_3_N_4_ has good thermal stability and can maintain its basic structure at medium-high temperatures (< 700 °C). However, the stability between g-C_3_N_4_ layers is reduced at medium-high temperature, which can cause destruction of the force between the layers. Thus, thermal oxidation treatment has been widely applied for the exfoliation of bulk g-C_3_N_4_. Moreover, the required processing time for thermal oxidation treatment to prepare high-surface-area g-C_3_N_4_ is shorter than that for liquid-phase ultrasound [11,23,69,79]. For example, g-C_3_N_4_ nanosheets with a specific surface area of 306 m^2^ g^−1^ and a thickness of 2 nm were prepared by thermal oxidation treatment of bulk g-C_3_N_4_ in air (at 500 °C for 2 h with 5 °C min^−1^) [69]. The average hydrogen evolution rate of the g-C_3_N_4_ nanosheets under UV-visible light was 170.5 μmol h^−1^, which is 5.4 times higher than that of the bulk g-C_3_N_4_. This can be ascribed to the synergistic effects of large surface area, increased bandgap, improved electron transport ability, and prolonged lifetime of the charge carriers. Additionally, the quality of the obtained material is inversely proportional to the processing time (Figure 7(c_1_)), because part of the g-C_3_N_4_ is oxidized to produce gas. In order to facilitate the exfoliation of g-C_3_N_4_, numerous studies have attempted to improve thermal oxidation treatment methods. Inspired by the preparation of highly corrugated graphene sheets, the high-surface-area g-C_3_N_4_ has been obtained by combining a rapid cooling process with thermal oxidation treatment [73,80]. Zhang et al. prepared g-C_3_N_4_ nanosheets (60.51 m^2^ g^−1^) via high-temperature treatment and a subsequent cooling process [80], heating the bulk g-C_3_N_4_ at 800 °C for 15 min with a heating rate of 600 °C min^−1^, then putting it in water to cool (15 °C). In our previous work, g-C_3_N_4_ nanosheets (142.8 m^2^ g^−1^) were prepared by rapid cooling after the heating process [73]. During the process of formation, the inter-layer force was broken by the shrinkage force generated from the rapid and large temperature difference, causing the gradual exfoliation of the g-C_3_N_4_. In addition, Zhang et al. synthesized g-C_3_N_4_ nanosheets (117.27 m^2^ g^−1^) by a continuous heating process, preparing the nanosheet structure with a smaller pore size by extending the thermal treatment time [74].

However, the degree of exfoliation in a single batch of samples may be different due to uneven heating in the thermal oxidation treatment. Therefore, bulk g-C_3_N_4_ is usually treated in small quantities to ensure uniform heating, resulting in a low yield. To solve this, Zhao et al. prepared g-C_3_N_4_ nanosheets with a high specific surface area (increasing from 10.94 m^2^ g^−1^ to 99.73 m^2^ g^−1^) by combining thermal oxidation treatment with liquid-phase ultrasound [81]. Reheating the dispersed g-C_3_N_4_ treated by ultrasound can avoid the occurrence of uneven heating, thus improving the yield of nanosheets. In addition to low yield, the application of thermal oxidation treatment for exfoliating bulk g-C_3_N_4_ has been limited by the single morphology of prepared g-C_3_N_4_ nanosheets. Recently, numerous works have prepared nanosheets with porous structures, including increased duration of treatment [77,82], changes of atmosphere [83,84,85], and addition of reagents [13,86,87,88,89]. For example, Li et al. extended the heating time to 6 h for preparing ultrathin g-C_3_N_4_ nanosheets (277.98 m^2^ g^−1^) with abundant micropores and mesopores [77]. Firstly, the interlayer forces have been broken by thermal oxidized process, then some basic units have been etched by increasing the calcination time, leading the formation of nanosheets with porous structures (Figure 7(c_2_)). The presence of abundant porous structures exposes more active edges and provides a cross-surface diffusion channel. It can promote the separation and transfer of photo-carriers, which is beneficial to the improvement of photocatalytic activity. Huangfu et al. prepared ultrathin g-C_3_N_4_ by repeated use of this simple heating method [82]. In addition, Hou et al. prepared porous nanosheet g-C_3_N_4_ with a specific surface area of 114 m^2^ g^−1^ by injecting hydrogen during the heating treatment [83]. There were many N vacancies in the structure, which overcame the quantum size effect, resulting in the simultaneous small size and narrow band gap. g-C_3_N_4_ with a porous structure (196 m^2^ g^−1^) and C vacancies was prepared by thermal oxidation treatment in ammonia gas, which enhanced the capacities of mass transfer, charge transfer, and light absorption [84]. Furthermore, Song et al. prepared the porous g-C_3_N_4_ nanosheets (265.2 m^2^ g^−1^) by thoroughly mixing bulk g-C_3_N_4_ with potassium hydroxide before reheating treatment in the air [86]. In this process, the adding alkali reacted with the C atoms to produce a porous structure for accelerating mass transfer, charge transfer, and the exposure of active sites. Hu et al. synthesized bimodal porous g-C_3_N_4_ nanosheets with a specific surface area of 219 m^2^ g^−1^ by the same method, which exhibited an improved photocatalytic hydrogen evolution of 1900 µmol h^−1^ g^−1^ (8.6 times higher than that of bulk g-C_3_N_4_) [87].

Although thermal oxidation treatment can exfoliate the bulk g-C_3_N_4_ effectively, low yields still restrict its further application. Due to the decomposition of C_3_N_4_ at high temperature, the yield of obtained g-C_3_N_4_ decreases with an increase in heating time and temperature. Moreover, the effect of exfoliation is closely related to the synthesis details in the process, including temperature, heating speed, treatment time, dosage, and atmosphere (Table 6) [69,73,74,77,79,80,81,82,83,84,85,86,87]. Thus, precise control of the trade-off between specific surface area and yield of exfoliated g-C_3_N_4_ still needs to be developed. Recently, thermal oxidation treatment has been combined with other methods to prepare g-C_3_N_4_ with a larger surface area.

##### Post-Hydrothermal

As one of the common methods used in preparation and treatment of materials, the hydrothermal method (solvothermal method) has also been applied in the post-processing of g-C_3_N_4_. Bulk g-C_3_N_4_ is usually placed in solution for subsequent hydrothermal treatment [12,70,75,76,90]. During this process, the presence of alkali can promote the formation of a porous structure and the surface modification of g-C_3_N_4_. Nie et al. prepared g-C_3_N_4_ with a fluffy porous structure (64.7 m^2^ g^−1^) via an alkali hydrothermal method [75]. The NO photocatalytic removal activity can be attributed to the increased specific surface area, narrow band gap, and low carrier recombination rate. However, the porous structure can be excessively enlarged or even destroyed by high concentrations of sodium hydroxide. In addition, ammonium hydroxide has been used in hydrothermal treatment of bulk g-C_3_N_4_ [70]. The C atoms can be partial oxidized by ammonium hydroxide, causing the formation of -OH and -C=O- on the surface of g-C_3_N_4_, which can promote the interaction of photocatalyst and reactant (Figure 7d). Moreover, the substitution of N is beneficial to the redox ability on the surface reaction sites. However, the excessive use of ammonium hydroxide can also cause structural damage, increasing the presence of sites for the recombination of photo-generated carriers. Wang et al. speculated that the proton exchange between -OH and -NH/NH_2_ affected the electron properties when the alkali concentration was too high and then inhibited the photocatalytic reaction [90]. Obviously, the main roles of hydrothermal method in treating bulk g-C_3_N_4_ are to promote the formation of the pore structure and surface modification. The concentration of alkali in the hydrothermal process needs to be explored to avoid excessive destruction of the g-C_3_N_4_ structure (Table 7) [70,75,76,90,91,92].

Recently, this method has been combined with other treatments to exfoliate bulk g-C_3_N_4_, which can make up for the drawbacks of the hydrothermal process, such as excessive defects and small specific surface area. For example, Zeng et al. prepared sea-urchin-structure g-C_3_N_4_ by combining sealed condensation with hydrothermal treatment [91]. The porous g-C_3_N_4_ was prepared by partial decomposition of g-C_3_N_4_ during the sealed condensation. Subsequently, this particular structure with a specific surface area of 65.6 m^2^ g^−1^ was formed by hydrothermal treatment. The sea-urchin-structure g-C_3_N_4_ showed a narrow band gap of 2.0 eV, overcoming the quantum size effect. Most importantly, its conduction band and valence band potential were suitable to promote overall water splitting under visible light irradiation. Additionally, the alkali hydrothermal process can be combined with carbon thermal reduction to optimize the morphology and electronic configuration of g-C_3_N_4_ [92]. The surface hydroxylation and exfoliation of g-C_3_N_4_ were performed via the former, and the electron configuration was optimized via carbon thermal reduction. The prepared g-C_3_N_4_ (197 m^2^ g^−1^) had a remarkably improved photocatalytic hydrogen evolution rate of 246.2 μmol h^−1^ under visible light irradiation (λ > 420 nm), 17-fold higher than that of bulk g-C_3_N_4_.

The above post-processing methods used in exfoliation of g-C_3_N_4_ are still restricted by the excessive defects (chemical exfoliation), long processing times (ultrasonication), low yield (thermal oxidation treatment), and structural damage (post-hydrothermal method). Gradually, the combination of two different methods has become a common pattern for the exfoliation of bulk g-C_3_N_4_ to make up for the shortcomings of single exfoliation methods. However, the details of the combinations between these methods need to be further explored. Furthermore, the entire design between band structure and morphological control of g-C_3_N_4_ should be considered to realize the excellent photocatalytic ability of g-C_3_N_4_.

### 2.4. Summary

**Figure 8:**
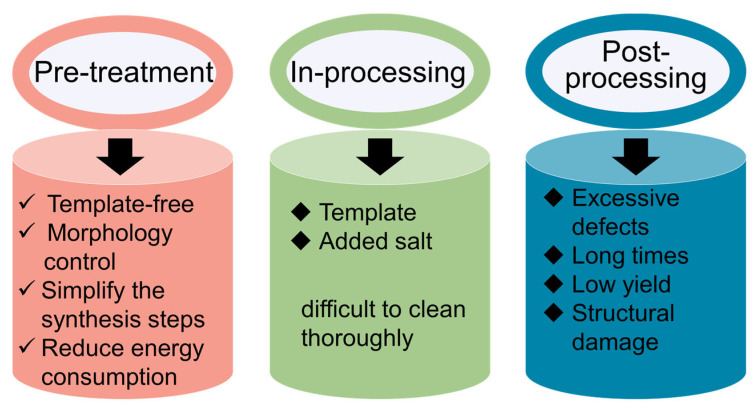
The characteristics of pre-treatment, in-processing, and post-treatment methods for the preparation of high-SSA g-C_3_N_4_.

**Figure 9:**
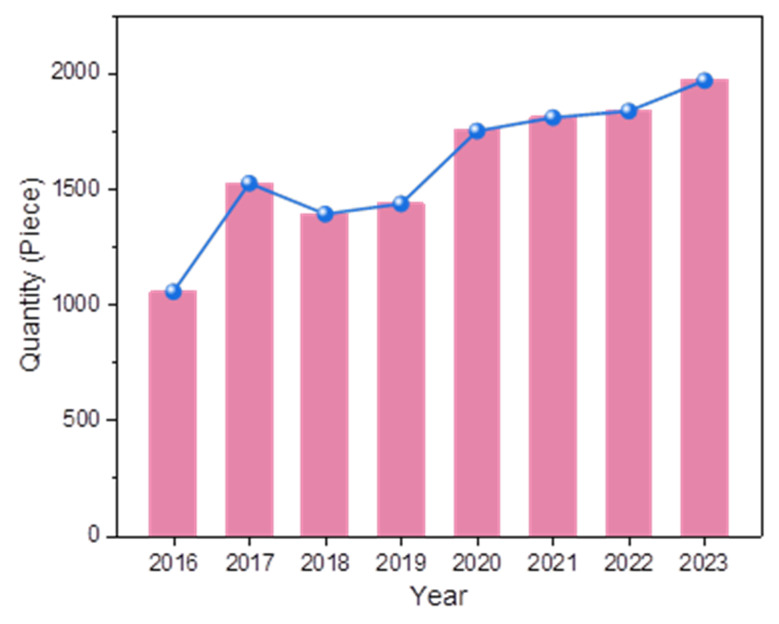
Number of articles on precursor treatment strategies from 2016 to 2023.

In summary, precursor mixing has emerged as a versatile and sustainable strategy for template-free synthesis of morphologically diverse g-C_3_N_4_ architectures with enhanced SSA. This approach circumvents the need for energy-intensive processes, toxic template removal, or additional reagents, offering inherent advantages in cost-effectiveness and environmental compatibility (Figure 8). As evidenced by the steadily rising publication trend in related studies from 2016 to 2023 (Figure 9), this methodology has garnered significant attention as a mainstream paradigm for engineering g-C_3_N_4_ with tailored nanostructures.

**Figure 10:**
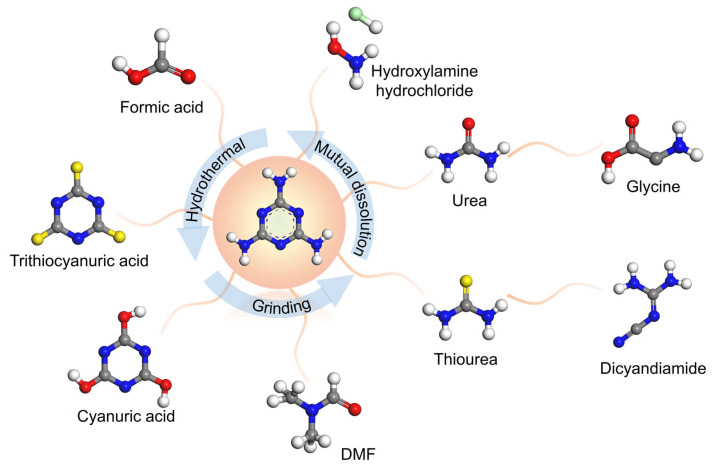
Precursors and treatment methods commonly used in precursor mixing strategies.

The mechanistic foundation of this strategy lies in the supramolecular preorganization of precursor complexes, where the selection and combinatorial principles of nitrogen-rich precursors dictate the self-assembly pathways. As illustrated in Figure 10, commonly employed precursors include melamine–cyanuric acid systems, urea–thiourea hybrids, doped precursors like Cu–melamine complexes; typical mixing modalities involve solvent-mediated hydrogen bonding, stoichiometry-controlled co-crystallization, and pH-triggered electrostatic assembly.

Crucially, the interplay between precursor chemistry (e.g., hydrogen-bonding capacity, π–π stacking propensity) and processing parameters (molar ratios, solvent polarity) governs the formation of intermediate supramolecular architectures, which template the final g-C_3_N_4_ morphology during thermal condensation. For instance, melamine–urea systems achieve nanotube formation through temperature-dependent cyanurate intermediate assembly, while acid-mediated precursor protonation induces lamellar exfoliation. Systematic mapping of these relationships, through combinatorial precursor screening coupled with machine learning-driven optimization, could enable predictive design of g-C_3_N_4_ catalysts with application-optimized SSA and defect landscapes.

## 3. Research Distribution and Trend Analysis of Carbon Nitride Applications

**Figure 11:**
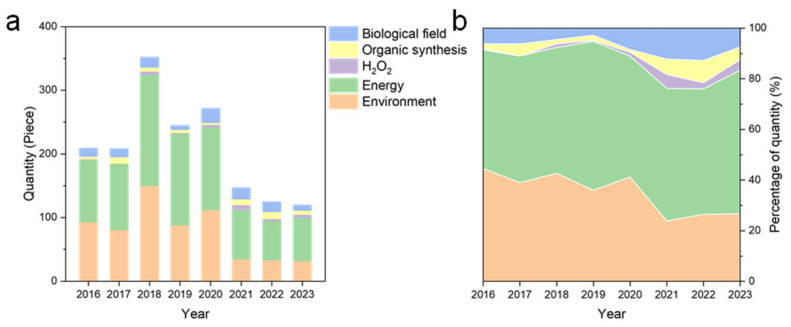
Statistical graph of carbon nitride applications in various fields (numbers of publications using “g-C_3_N_4_” or “carbon nitride” as topic keywords since 2016), (a): quantity; (**b**): percentage of quantity.

**Figure 12:**
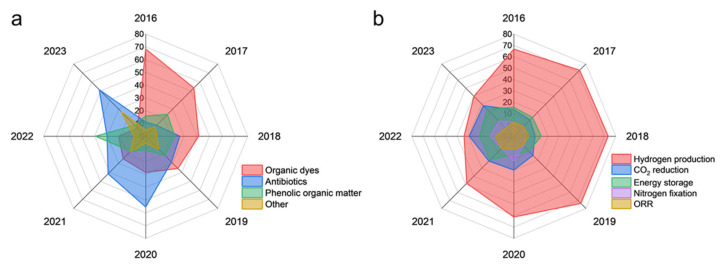
Annual distribution map of articles published in the fields of (**a**) degradation of pollutants in water and (**b**) energy (numbers of publications using “g-C_3_N_4_” or “carbon nitride” as topic keywords since 2016).

The escalating environmental pollution and energy crises demand transformative solutions, driving the exploration of advanced semiconductor materials. Among them, g-C_3_N_4_ has emerged as a versatile platform due to its tunable electronic structure, visible-light responsiveness, and earth-abundant composition. Our bibliometric analysis (Figure 11a) reveals a declining trajectory in g-C_3_N_4_ research across traditional domains (2016–2023), with energy and environmental applications dominating ~80% (decreasing from 90%-2019 to 80%-2023) of publications (Figure 11b). Notably, emerging trends highlight a shift to in environmental field from dye degradation (60% decline since 2016) to emerging refractory pollutants (antibiotics: +40%) (Figure 12a), a transition in the energy sector from photocatalytic H_2_ production (−30% since 2016) to CO_2_ reduction (+20%) (Figure 12b), and steady growth in antibacterial and biosensing research for biomedical applications (~10% of total) (Figure 11b). This evolution reflects the material’s adaptability to address sustainability imperatives and technological bottlenecks. The following sections dissect these trends with mechanistic insights and case studies.

### 3.1. Environmental Remediation: From Conventional Pollutant Removal to Emerging Contaminants

g-C_3_N_4_-based systems excel in multiphase pollutant management through light-driven redox cycles (Table 8) [21,32,33,37,39,43,49,62,73,79,83,93,94,95,96,97]. The photocatalytic mechanism involves photon absorption (λ > 420 nm for visible-light activation), carrier generation and migration (e^−^-h^+^ pairs with CB~−1.3 V vs. NHE), formation of reactive species (•OH, •O_2_^−^ via H_2_O/O_2_ activation), and pollutant mineralization (e.g., aromatic ring cleavage in antibiotics). Furthermore, a high SSA directly enhances remediation efficacy through the enlarged pollutant adsorption capacity and the increased density of active sites for redox reactions.

#### 3.1.1. Aquatic Organic Contaminant Degradation

Dyes, nitrogen-containing organic compounds, phenols, and antibiotics are often used as target pollutants to test the degradation ability of g-C_3_N_4_ materials [85,98,99,100,101]. While early studies focused on model organic dyes (e.g., methylene blue), recent efforts have targeted priority pollutants. For example, the photodegradation activity of polyaniline (PANI)/carbon nitride nanosheet (CNNS) composite hydrogel was tested via the degradation of methylene blue. The excellent performance in removing organic pollutant can be ascribed to the cooperation of adsorptive preconcentration and the subsequent photocatalytic oxidation [98]. Zhang et al. constructed a Bi_7_O_9_I_3_/C_3_N_4_ Z-scheme heterojunction photocatalyst for degrading doxycycline hydrochloride under visible light [99]. The dominant oxhydryl and superoxide active groups led to excellent photodegradation and mineralization ability for doxycycline hydrochloride. Moreover, the prepared tetragonal carbon nitride hollow tubes exhibited superior photodegradation activities for methylene blue and phenol thanks to the content of nitrogen impurities nitrogen, a unique hollow structure, and a larger specific surface area; porosity directly correlated with reaction kinetics [43]. The combination of advanced oxidation processes and photocatalysis can further enhance the photodegradation activity of g-C_3_N_4_. An et al. reported a single-atom Fe g-C_3_N_4_ catalyst with Fe (II)-Nx active sites to accelerate the production of HO· radicals, leading to excellent degradation efficiency for various organics (MB, MO, RhB, and phenol) [100].

#### 3.1.2. Heavy Metal Detoxification

Toxic heavy metal ions in industrial wastewater can also be removed by g-C_3_N_4_’s dual adsorption-photoreduction capability [102,103,104,105]. In order to enhance the charge migration, Lei et al. coupled carbon nitride nanosheets with MIL-88B(Fe), which possessedthe highest catalytic activity to reduce Cr (VI) under visible light [105]. Three-dimensional g-C_3_N_4_@cellulose aerogel is certified to remove hexavalent chromium and antibiotics simultaneously under light irradiation, where hierarchical pores enable simultaneous adsorption and reduction [106]. Pd nanocones supported on g-C_3_N_4_ showed enhanced catalytic reduction of hexavalent chromium under visible-light irradiation [107].

#### 3.1.3. Air Purification

The removal rate of NO commonly used to investigate the degradation capacity of C_3_N_4_ in air [108,109,110,111,112]. Li et al. prepared an illite particle-modified g-C_3_N_4_ to enhance the photocatalytic NO removal activity of g-C_3_N_4_ [113]. The formation of heterojunctions between illite and g-C_3_N_4_ accelerated the charge migration, leading toexcellent photocatalytic NO removal activity. Porous g-C_3_N_4_ microtubes with N-vacancies showed 2.6 times higher NO removal than bulk material, and the 3D porous wall structure facilitated gas diffusion and exposure of active sites [39]. The existence of N-vacancies can improve the adsorption ability for NO, the charge capture, and the light-absorbing capability of g-C_3_N_4_. Meanwhile, the diffusion of reactants and the transfer of charge are promoted by the porous wall structure. In addition, the preparation of 3D materials is considered an efficient approach for air purification. Hu et al. modified g-C_3_N_4_ with perylene imide (PI) and graphene oxide (GO) to prepare a g-C_3_N_4_-based aerogel [112]. The excellent activity in NO removal that was observed was attributed to the strong light absorption and favorable charge transport.

#### 3.1.4. Outlook

In the environmental research, the application of g-C_3_N_4_ materials in degrading organic pollutants in water is still a popular research direction. It is worth noting that the targets of degradation have shifted from organic dyes to emerging refractory pollutants (antibiotics, phenolic organic matter) (Figure 12a). Nonetheless, there are still many challenges relating to the practical application of g-C_3_N_4_ materials for the degradation of pollutants in water, such as the pathway and mechanism of degradation, limited real wastewater validation, and the trade-off between SSA enhancement and quantum efficiency loss. Future work must resolve this fundamental trade-off; SSA expansion often introduces defect-induced recombination centers, demanding balanced design to preserve quantum efficiency.

### 3.2. Energy Conversion and Storage: Bridging Photocatalysis to Practical Energy Systems

It is well known that the photo-generated electrons of g-C_3_N_4_ have a certain reduction ability due to the location of the appropriate conduction band, which can be adjusted by various approaches. Since they were found to be able to produce hydrogen, g-C_3_N_4_ materials have been widely used in many fields of energy regeneration, including hydrogen production, CO_2_ reduction, nitrogen fixation, and so on (Table 9) [25,26,28,36,38,46,51,53,54,55,58,60,65,69,71,74,77,81,82,84,114,115,116,117,118,119]. Enhanced SSA improves energy conversion efficiency by exposing more catalytic sites, shortening charge migration paths, and facilitating mass transport.

#### 3.2.1. Solar Hydrogen Production

As the most ideal renewable energy, hydrogen energy has the advantages of clean, efficient, and convenient storage. Photocatalytic water splitting technology is a commonly used green method for hydrogen production. The reaction process is shown in the following equation:

(2) $$4H^{+}+4e^{-}\to 2H_{2}$$

(3) $$H_{2}O+2h^{+}\to O_{2}+2H^{+}+4e^{-}$$

The precondition of photocatalytic reaction is that the potential of the valence band in the catalyst is more positive than that of O_2_/H_2_O (1.23 V vs. NHE), and its potential of conduction band is more negative than that of H^+^/H_2_ (0 V vs. NHE). In the past few years, g-C_3_N_4_ has been widely used in photocatalytic hydrogen production, due to its suitable conduction band [28,120,121,122]. The purpose of modification is to increase the number of photogenerated electrons in the system to support the reduction reaction. Hollow g-C_3_N_4_ nanotubes with an eight-fold increase in specific surface area prepared by precursor mixing were applied to product hydrogen with Pt as the cocatalyst, and exhibited a higher photocatalytic production rate of 1073.6 µmol·h^−1^·g^−1^ [28]. The enhanced photocatalytic H_2_ production benefited from the porous and nanotubular structure, which facilitated charge carrier migration and separation. Furthermore, Che et al. enhanced the overall water splitting of g-C_3_N_4_ by preparing an in-plane (C ring)-C_3_N_4_ heterostructure with fast spatial transfer of photo-generated electrons [122]. Single-atom loading is an efficient approach to further enhance the hydrogen production rate of g-C_3_N_4_. For example, single-atom Cu decorated tubular g-C_3_N_4_ exhibited a superior visible-light photocatalytic hydrogen production rate (≈212 µmol h^−1^/0.02 g catalyst), which was ascribed to the improved in-plane and interlayer separation/transfer of the charge carriers [123].

#### 3.2.2. CO_2_ Photoreduction

Recently, photocatalytic technology has been applied in CO_2_ photoreduction to produce hydrocarbon fuels. This technology has become a research hotspot due to its bright prospects for alleviating the greenhouse effect and energy crisis simultaneously. The basic steps include the adsorption of CO_2_, the production and migration of carriers, the reduction reaction, and the desorption of products. The main evaluation parameters of CO_2_ photoreduction performance are conversion rate and selectivity.

As a hot semiconductor, g-C_3_N_4_ has been considered a potential catalyst for CO_2_ reduction [124,125,126,127]. Nevertheless, the activity of bulk g-C_3_N_4_ in CO_2_ reduction remains inhibited by their small specific surface area, high recombination of photo-generated carriers, and low electron conductivity. A 3D porous g-C_3_N_4_/C nanosheets composite was successfully prepared to achieve the excellent CO_2_ reduction activity [128]. Owing to its enhanced light trapping/utilization, highly efficient CO_2_ adsorption ability by mesopores, and low recombination of photogenerated carriers, it showed better CO_2_ reduction activity than that of bulk g-C_3_N_4_ (CO and CH_4_ yield of 229 and 112 μmol g^−1^-cat). Moreover, single Ni atoms have been anchored on porous few-layer g-C_3_N_4_ to improve photocatalytic CO_2_ reduction [129]. Thanks to the synergistic N-Ni-N connection and interfacial carrier transfer, Ni-C_3_N_4_ exhibited a CO generation rate of 8.6 µmol g^−1^ h^−1^ under visible light illumination, which was 7.8 times that of the porous few-layer g-C_3_N_4_ (1.1 µmol g^−1^ h^−1^). Samanta et al. enhanced the photocatalytic CO_2_ reduction of g-C_3_N_4_ by preparing doped g-C_3_N_4_ [124]. The C, O co-doped g-C_3_N_4_ exhibited a higher yield of CH_3_OH (4.18 mmol g^−1^ in 6 h) than bulk C_3_N_4_ (2.8 mmol g^−1^).

#### 3.2.3. Nitrogen Fixation

Nitrogen fixation, a potential pathway for ammonia synthesis, has attracted wide attention in the field of energy as a solution for the energy and environmental problems caused by traditional ammonia synthesis. The whole process is a multi-step reaction between photo-generated electrons and protons.

(4) $$H_{2}O\to \frac{1}{2}O_{2}+2H^{+}+2e^{-}$$

(5) $$2H^{+}+2e^{-}\to H_{2}$$

(6) $$N_{2}+e^{-}\to 2N_{2}^{-}$$

(7) $$N_{2}+H^{+}+e^{-}\to N_{2}H$$

(8) $$N_{2}+2H^{+}+2e^{-}\to N_{2}H_{2}$$

(9) $$N_{2}+4H^{+}+4e^{-}\to N_{2}H_{4}$$

(10) $$N_{2}+5H^{+}+4e^{-}\to N_{2}H_{5}^{+}$$

(11) $$N_{2}+6H^{+}+6e^{-}\to 2NH_{3}$$

(12) $$N_{2}+8H^{+}+6e^{-}\to 2NH_{4}^{+}$$

It can be seen that this process requires a large number of electrons, competes with hydrogen production, and involves intermediates and by-products. Despite thermodynamic challenges (N≡N bond dissociation: 941 kJ mol^−1^), some advances have been made [130,131,132]. Qiu et al. combined black phosphorus nanosheets with the g-C_3_N_4_ nanosheets to enhance performance in visible-light nitrogen photo-fixation [132]. The formation of C-P covalent bonds increased the number of excited electrons and facilitated the separation efficiency of carriers. Additionally, the obtained ultrathin sulfur-doped g-C_3_N_4_ porous nanosheets with large lateral size and carbon vacancies had a photocatalytic nitrogen fixation rate of 5.99 mMh^−1^ gCat^−1^ (2.8 times higher than that of bulk g-C_3_N_4_) due to the carbon vacancies and edge sites synergizing for N₂ activation [133]. Yin et al. synthesized single-atom electrocatalysts consisting of transition metal atoms and monolayer g-C_3_N_4_ for NH_3_ production [134]. For single-atom Pt-C_3_N_4_, the adsorption capacity of N_2_ is higher than that of H atoms, suggesting excellent nitrogen-fixation activity.

#### 3.2.4. Energy Storage

In addition to the above, g-C_3_N_4_-based composites have been applied in energy storage [135,136,137,138]. For instance, Wang et al. introduced g-C_3_N_4_ into a three-dimensional hierarchical porous graphene to achieve excellent cycling performance for sulfur cathodes in lithium–sulfur batteries [137]. In lithium-ion batteries, the prepared layered g-C_3_N_4_@reduced graphene oxide composites possessed excellent cycle stability (899.3 mAh g^−1^ after 350 cycles under 500 mA g^−1^) and remarkable rate performance (595.1 mAh g^−1^ after 1000 cycles under 1000 mA g^−1^), because of the large interlayer distances, rich N-active sites, and a microporous structure accommodating volume expansion [138]. Zhang et al. prepared a 3D porous sulfur/graphene@g-C_3_N_4_ hybrid sponge and used it as a cathode for Li-S batteries [135]. The superior high-rate capability (612 mAh g^−1^ at 10 C) benefited from the numerous adhesion sites of polysulfides and the efficient electron/Li^+^ transport pathways.

#### 3.2.5. Outlook

As shown in Figure 12b, the research on CO_2_ reduction and nitrogen fixation in addition to hydrogen production is increasing gradually. The further development of these directions is of great significance to alleviate the energy crisis. In order to prepare g-C_3_N_4_ materials with excellent performance, further exploration of reaction mechanisms and pathways is necessary. While CO_2_RR and N_2_ fixation show promise, scalability requires breakthroughs in catalyst stability under continuous operation and product-separation technologies for mixed-output systems. Scalability requires stabilizing high-SSA architectures against restructuring during continuous operation.

### 3.3. Biomedical Innovations: Antimicrobial and Sensing Platforms

#### 3.3.1. Photodynamic Antimicrobial Therapy

There are many microorganisms which may be harmful to the human body. The antibacterial process uses the active substances produced by photocatalyst, such as superoxide anion and hydrogen peroxide, to inhibit the reproduction of microorganisms and hinder their internal reactions. Therefore, the antibacterial properties of g-C_3_N_4_ materials depend on its ability to produce active substances [139,140,141,142]. g-C_3_N_4_ nanosheets have attached increasing attention for use in photocatalytic disinfection, due to their enhanced optical, mechanical, and electrical properties. Li et al. assembled functionalized g-C_3_N_4_ nanosheets composite membranes with superior self-cleaning and antimicrobial properties [140]. Kang et al. developed high-quality g-C_3_N_4_ nanosheets with 82.61 m^2^/g via a bacterial etching approach, obtaining better photocatalytic disinfection performance compared with bulk g-C_3_N_4_ [139]. In addition, a g-C_3_N_4_/perylene-3,4,9,10-tetracarboxylic diimide (PDINH) heterostructure was synthesized to increase the number of reactive oxygen species, leading to an excellent inactivation effect on bacteria [141].

#### 3.3.2. Biosensing and Diagnostics

Another application of g-C_3_N_4_ materials in the field of biology is photo-electrochemical sensing, which depends on visible-light response and the efficiency of charge carrier separation [63,143,144,145,146]. For example, the core–shell LaFeO_3_@g-C_3_N_4_ p-n heterostructure showed improved photoelectrochemical performance for streptomycin sensing, due to the wider absorption band edge and stronger photocurrent signal [146]. Ultrathin g-C_3_N_4_ nanosheets have been used as heparin sensing platform with a heparin detection limit of 18 ng mL^−1^ [63]. In addition, Chen et al. tested DNA methyltransferase activity using a g-C_3_N_4_ nanosheet electrochemiluminescence biosensor, which exhibited an ultralow detection limit down to 0.043 U mL^−1^ [143].

#### 3.3.3. Outlook

It can be seen that the wide application of g-C_3_N_4_ materials in the biological field can bring great benefits to humans in terms of medical and health care. However, several challenges persist. These include concerns over biocompatibility in in vivo applications, the paucity of research on long-term cytotoxicity, and the difficulties associated with the scalable synthesis of medical-grade g-C_3_N_4_. Increased SSA enhances biomedical functionality by amplifying reactive oxygen species (ROS) generation per unit mass and improving biomolecule adhesion.

## 4. Conclusions and Outlook

The progress in research into g-C_3_N_4_ with large specific surface areas has been summarized and discussed in this work, involving the applicationand controllable synthesis of high-SSA g-C_3_N_4_. Several applications of g-C_3_N_4_ materials have been presented. It can be seen that the research into g-C_3_N_4_ has involved many fields relating to energy and the environment, including hydrogen production, degradation, energy storage, sensing, and so on. Nevertheless, the application of g-C_3_N_4_ in some fields has not been deeply explored. Thus, g-C_3_N_4_ is still a potential material to mitigate energy and environmental crises. Although the performance of g-C_3_N_4_ has significantly improved in recent years, there are still challenges to be addressed in the exploration of g-C_3_N_4_.

(i) For the design and synthesis of g-C_3_N_4_ materials with excellent performance, overall consideration should be given to the specific surface area, band structure, and defects of g-C_3_N_4_ materials. For photocatalysis, the realization of excellent photocatalytic efficiency requires the combined efforts of the light absorption ability, charge transfer, and separation ability, and adsorption ability for the catalyst’s reactants. Mechanisms to promote the properties of g-C_3_N_4_-derived materials should be explored. The further functionalization mentioned in this work can improve the performances of g-C_3_N_4_ by promoting the transfer and separation of charge. However, the exact transfer pathway of charge in g-C_3_N_4_ derived material is still ambiguous or unsubstantiated. Further exploration of the mechanism is of great significance for the synthesis of g-C_3_N_4_ materials with target structures. Combination of improved methods is required. The published methods for modification of g-C_3_N_4_ have usually aimed to improve a particular aspect of performance. Therefore, the synthesis of high-performance g-C_3_N_4_ can be realized by combining various modification methods according to the requirements of specific application fields. The design and assembly of material reactors should be considered. Generally, most prepared g-C_3_N_4_ materials are powder, which is not suitable for all applications. The design of suitable reactors can meet the particular requirements of some reactions to broaden the application range of g-C_3_N_4_.
(ii) Further exploration of reaction mechanisms should include interface reactions between reactants and g-C_3_N_4_ materials. Many applied reactions of g-C_3_N_4_ materials have occurred in water or other media where the reaction isdirectly affected by the interface effect between reactants and materials. Regarding the pathways of g-C_3_N_4_ material reactions with reactants, our experimental process was a macroscopic process, so that the specific reaction path between g-C_3_N_4_ materials and the reactants could not be fully assessed. Such exploration is very important for the targeted synthesis and application of high-performance g-C_3_N_4_ materials. Theoretical calculations related to g-C_3_N_4_ materials can be combined with experimental results to clarify the specific active mechanisms of g-C_3_N_4_ materials, which is conducive to understanding the properties of materials and the occurrence of reactions. However, the direction of calculation needs to be further explored.
(iii) Application fields of g-C_3_N_4_ materials include practical industrial application of g-C_3_N_4_ materials in “old” fields. The degradation activity of the g-C_3_N_4_ material under actual water conditions should be studied, including the coexistence of various pollutants, fluid medium, sunlight irradiation, and so on. With regard to improving the performances of g-C_3_N_4_ material in “new” fields, g-C_3_N_4_ materials have been subject to a relatively few studies in the fields of nitrogen fixation, antibacterial activity, and sensing, leaving large room for improvement.

Thus, g-C_3_N_4_ materials with target performances and structures can be designed based on the reaction mechanism to realize their value in the fields of energy and the environment in the future.

## Figures and Tables

**Table 1:** Influencing factors and raw materials [20,21,25,26,27,28,29].

References	Precursor 1	Precursor 2	Mixing Mode	Calcining Process	Results			
					Bulk g-C_3_N_4_ BET	Increased BET	Morphology	Application
[28]	Melamine 0.5 g	Urea 5 g	-	550 °C for 4 h 5 °C min^−1^ in a nitrogen	5.6 m^2^/g	42.2 m^2^/g	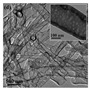	Photocatalytic hydrogen evolution
[21]	Cyanuric acid 2.58 g	Melamine 2.52 g	Stirred for 2 h	550 °C for 4 h	17.74 m^2^/g	81.58 m^2^/g	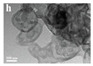	H_2_ production activity and degradation rate
[25]	5 g of formic acid	3 g of melamine	Hydrothermal treatment	550 °C for 4 h	-	81.4 m^2^/g	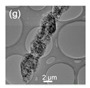	Photocatalytic hydrogen evolution
[20]	1.0 g melamine	2.0 g hydroxylamine hydrochloride	Hydrothermal process	520 °C for 4 h in air	3.9 m^2^/g	129.4 m^2^/g	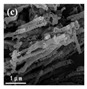	Photocatalytic H_2_O_2_ production
[26]	4 g of melamine	50 mL of N,N- dimethylformamide	Fully mixed at 25 °C for 0.5 h	550 °C for 4 h under air	11.23 m^2^/g	181.74 m^2^/g	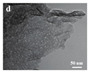	Photocatalytic hydrogen evolution
[27]	Melamine	Cyanuric acid Phosphorous acid	Hydrothermal process	520 °C for 4 h	-	-	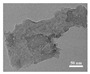	Electrochemiluminescence
[29]	0.01 mol melamine	0.01 mol cyanuric acid	Stirred for 12 h at room temperature	550 °C for 4 h 5 °C min^−1^	10.83 m^2^/g	130 m^2^/g	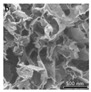	Photocatalytic overall water splitting

**Table 2:** The influencing factors and results of precursor pretreatment [32,33,34,35,36,37,38,39,40,42,43].

References	Precursor	Added Reagent	Treatment Mode	Calcining Process	Results			
					Bulk g-C_3_N_4_ BET	Increased BET	Morphology	Application
[32]	Melamine	1 mL HCl (37%)	In 80 mL of hot distilled water Stirring for 30 min	500 °C for 2 h 20 °C/min 520 °C for 2 h	8.5 m^2^/g	345 m^2^/g	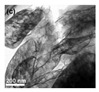	Photocatalytic activity for NO removal
[33]	Melamine	3 mL concentrated HCl (1:1, *v*/*v*)	In 30 mL of absolute alcohol Stirring for 30 min	550 °C for 4 h	12.7 m^2^/g	26.2 m^2^/g	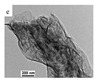	Photocatalytic degradation
[34]	Melamine	0.6 M HNO_3_ solution (50 mL)	50 mL of ethylene glycol Stirring at room temperature	550 °C for 2 h	16.6 m^2^/g	86.4 m^2^/g	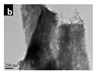	Photocatalytic hydrogen evolution
[35]	Melamine	H_2_SO_4_:H_2_O 1:1 in volume	In distilled water (100 mL) Stirring for 2 h	380 °C in 5 min 600 °C for 4 h 1 °C min^−1^ in Ar	8.6 m^2^/g	15.6 m^2^/g	-	Photocatalytic hydrogen evolution
[36]	Urea	50 mL of methanol	Diethyl until white jellylike crystallization occurred	600 °C for 2 h 2.3 °C min^−1^ in Ar	43.1 m^2^/g	228.4 m^2^/g	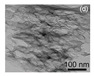	Photocatalytic hydrogen evolution
[37]	Melamine	Dried dimethyl sulfoxide 100 mL	180 °C under magnetic stirring	-	7.94 m^2^/g	669.15 m^2^/g	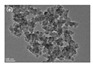	Photocatalytic degradation
[42]	Dicyandiamide	NH_4_Cl	Frozen in liquid nitrogen	550 °C for 4 h 3 °C min^−1^ in N_2_	-	65 m^2^/g	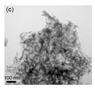	Photocatalytic hydrogen evolution
[43]	Melamine	352 °C for LiCl-KCl	Milled together	450 °C for 5 h 4 °C min^−1^ in air	7 m^2^/g	128 m^2^/g	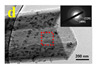	Photocatalytic degradation
[38]	Melamine	Deionized water (40 mL)	200 °C for 12 h	550 °C for 4 h 2 °C min^−1^	8.6 m^2^/g	127.8 m^2^/g	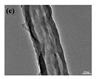	Photocatalytic hydrogen evolution
[39]	Melamine	Deionized water (70 mL)	180 °C for 12 h	550 °C for 3 h 2.5 °C min^−1^	19.9 m^2^/g	67.5 m^2^/g	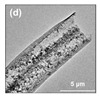	Photocatalytic activity for NO removal
[40]	Dicyandiamide	Deionized water (65 mL)	200 °C for 2 h	550 °C for 4 h 5 °C min^−1^	12.2 m^2^/g	59.8 m^2^/g	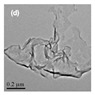	Photocatalytic hydrogen evolution

**Table 3:** Influencing factors and results of template methods [44,45,46,47,48,49,50,51,52,53,54,55,56,57,58].

References	Template	Precursor	Mixing Mode	Calcining Process	Removing Template	Results			
						Bulk g-C_3_N_4_ BET	Increased BET	Morphology	Application
[44]	SBA-15	Ethane diamine CCl_4_	Refluxed and stirred at 90 °C for 6 h	600 °C for 5 h 3.0 °C min^−1^ in a nitrogen	5 wt. % hydrofluoric acid	-	505 m^2^/g	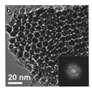	-
[45]	SBA-15 10.7 nm	Ethane diamine CCl_4_	Refluxed and stirred at 90 °C for 6 h	600 °C for 5 h 3.0 °C min^−1^ in nitrogen	5 wt. % hydrofluoric acid	-	830 m^2^/g	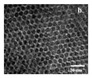	The Friedel-Crafts acylation of benzene
[46]	SBA-15	Cyanamide	Stirred for 1 h	550 °C for 4 h 2.3 °C min^−1^	NH_4_HF_2_ 4 M	-	239 m^2^/g	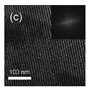	Photocatalytic Hydrogen Evolution
[47]	SBA-15	Ammonium thiocyanate	Stirred at 100 °C to remove water	550 °C for 2 h	NH_4_HF_2_ 4 M	9 m^2^/g	239 m^2^/g	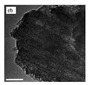	Photocatalytic Hydrogen Evolution
	SiO_2_						188 m^2^/g	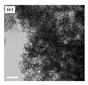	
[48]	SBA-15	Hexamethylene-tetramine	Stirred at room temperature	750 °C in nitrogen	40% of HF	-	1116 m^2^/g	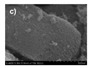	Dehydrogenation of ethylbenzene to styrene
[49]	SBA-15	Dicyandiamide	Vaporized at 70 °C	550 °C for 3 h	NH_4_HF_2_ 4 M	16.7 m^2^/g	50.1 m^2^/g	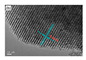	Photocatalytic degradation of fluoroquinolone antibiotics
[50]	MCM-22	Ethane diamine CCl_4_	Refluxed at 90 °C for 6 h	600 °C for 5 h 3.0 °C min^−1^ in nitrogen	5 wt. % hydrofluoric acid	less than 25 m^2^/g	739 m^2^/g	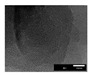	-
[51]	SiO_2_	Cyanamide	Stirred for 30 min (CA at 0.01 N HCl and ethanol pH 2, adding TEOS)	550 °C for 4 h 2.3 °C min^−1^ in argon	NH_4_HF_2_ 4 M	-	131 m^2^/g	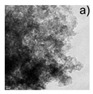	Photocatalytic Hydrogen Evolution
[52]	SiO_2_ 12 nm	Cyanamide	Stirred at 333 K for 12 h	823 K for 4 h 2.3 °C min^−1^ under N_2_	NH_4_HF_2_ 4 M	10 m^2^/g	160 m^2^/g 228 m^2^/g	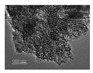	Photocatalytic H_2_O_2_ Production
[53]	Multishell SiO_2_ nanospheres	Cyanamide	Stirred at 40 °C for 8 h	550 °C for 3h under N_2_	Na_2_CO_3_ 0.3 M	-	310.7 m^2^/g	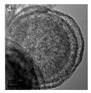	Photocatalytic Hydrogen Evolution
[54]	Chiral mesoporous SiO_2_ films	Cyanamide	Sonicated at 55 °C for 4 h	550 °C for 4 h 4 °C min^−1^ in N_2_	NH_4_HF_2_ 4 M	6.03 m^2^/g	132.26 m^2^/g	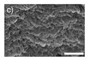	Photocatalytic Hydrogen Evolution
[55]	SiO_2_ nanotubes with porous shells	Cyanamide	Stirring for 10 min, separating, and drying (three times)	550 °C for 4 h in N_2_	10% of HF	4.6 m^2^/g	135.1 m^2^/g	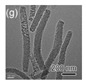	Photocatalytic Hydrogen Evolution
[56]	SiO_2_ microspheres	Melamine	In-air CVD method 320 °C for 2 h	550 °C for 3 h	NH_4_HF_2_ 4 M	10.1 m^2^/g	29.9 m^2^/g	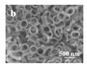	Photocatalytic Hydrogen Evolution
[57]	SiO_2_	Cyanamide	Stirred at 25 °C for 24 h NH_4_OH Stirred for about 5 min	550 °C for 4 h 1 °C min^−1^ in N_2_	NH_5_F_2_ 4 M	-	69.1 m^2^/g	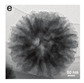	Photocatalytic Hydrogen Evolution
[58]	KCC-1	Cyanamide	Sonication at 55 °C for 4 h (HCl-treated KCC-1)	550 °C for 4 h	NH_4_HF_2_ 4 M	9 m^2^/g	160 m^2^/g	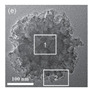	Photocatalytic Hydrogen Evolution

**Table 5:** Influencing factors, assistant methods, and results of ultrasonic processing [68,71,72,78].

References	Synthesis of Bulk g-C_3_N_4_	Ultrasonic Process			Assistant Method	Results			
		M_bulk g-C3N4_	Solvent/V_Solvent_	Treated Time		Band Gap	BET	Morphology	Application
[68]	Melamine 600 °C for 2 h 3 °C/min in air	0.1 g	Water 100 mL	16 h	-	2.64 eV ↓ 2.70 eV	-	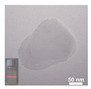	Bioimaging
[71]	Commercial g-C_3_N_4_	0.03 g	IPA 10 mL	10 h	-	2.35 eV ↓ 2.65 eV	384 m^2^/g	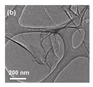	Hydrogen Evolution
[78]	Dicyandiamide 350 °C for 2 h 600 °C for 2 h	0.06 g	1,3-BUT 25 mL	24 h	-	2.65 eV ↓ 2.79 eV	3.3 m^2^/g ↓ 32.54 m^2^/g	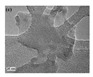	The sensor for trace amounts of Cu^2+^ determination Photocatalytic degradation
[72]	Melamine 550 °C for 4 h in static air 2.3 °C/min.	0.5 g	Ethanol–water 150 mL	10 h	-	2.70 eV ↓ 2.79 eV	12.5 m^2^/g ↓ 59.4 m^2^/g	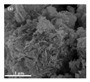	Photocatalytic degradation

**Table 6:** Influencing factors, assistant methods, and results of thermal oxidation treatment [69,73,74,77,79,80,81,82,83,84,85,86,87].

References	Synthesis of Bulk g-C_3_N_4_	Thermal Oxidation Treatment			Assistant Method	Results			
		M_bulk g-C3N4_	Temperature	Treated Time		Band Gap	BET	Morphology	Application
[69]	Dicyandiamide 550 °C for 4 h in static air 2.3 °C/min	0.4 g	500 °C 5 °C/min	2 h in static air	-	2.77 eV ↓ 2.97 eV	50 m^2^/g ↓ 306 m^2^/g	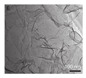	Photocatalytic hydrogen evolution
[79]	Thiourea 550 °C for 2 h 15 °C/min	-	550 °C	2 h in air	-	2.42 eV ↓ 2.86 eV	27 m^2^/g ↓ 151 m^2^/g	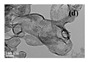	Visible light photocatalytic removal of NOx
[80]	Melamine 520 °C for 4 h 5 °C min^−1^ in static air	1.0 g	800 °C 600 °C min^−1^	15 min	cooled by circulation cooling water (15 °C)	2.64 eV ↓ 2.81 eV	7.38 m^2^/g ↓ 60.51 m^2^/g	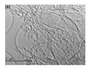	Photocatalytic hydrogen evolution
[73]	Dicyandiamide 550 °C for 4 h 2 °C/min in static air	0.5 g	500 °C 5 °C/min	2 h	put quickly into liquid nitrogen	2.68 eV ↓ 2.57 eV	15.6 m^2^/g ↓ 142.8 m^2^/g	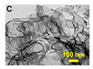	Photocatalytic degradation
[74]	Urea 550 °C for 9 h 10 °C/min in air	-	-	-	A single continuous heating process	-	40.22 m^2^/g ↓ 117.27 m^2^/g	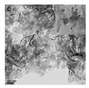	Photocatalytic hydrogen evolution
[81]	Melamine 520 °C for 4 h in air 5 °C/min	0.5 g	520 °C 5 °C/min	4 h in air	0.5 g 200 mL water ultrasonication 2 h	2.64 eV ↓ 2.72 eV	10.94 m^2^/g ↓ 99.73 m^2^/g	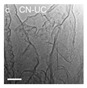	Photocatalytic hydrogen evolution
[77]	Melamine 773 K for 2 h 2 K min ^−1^ 793 K for 2 h	1 g	793 K 2 K min ^−1^	6 h	Increased calcination time	2.67 eV ↓ 2.81 eV	10.89 m^2^/g ↓ 277.98 m^2^/g	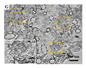	Photocatalytic hydrogen evolution
[82]	Melamine 550 °C for 4 h 2 °C min^−1^	0.4 g	550 °C	30 min	wice	-	-	-	Lithium-sulfur batteries
[83]	Dicyandiamide 550 °C for 4 h 5 °C min^−1^ in air	-	600 °C	2 h	H_2_ atmosphere	2.78 eV ↓ 1.82 eV	7 m^2^/g ↓ 114 m^2^/g	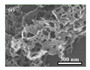	Photoeletrocatalytic Degradation of 4-Chlorophenol
[84]	Dicyandiamide 550 °C for 4 h	0.3 g	510 °C	1 h	NH_3_ atmosphere	2.59 eV ↓ 2.90 eV	6 m^2^/g ↓ 196 m^2^/g	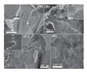	Photocatalytic hydrogen evolution
[83]	Melamine 550 °C for 4 h 5 °C/min in N_2_ gas	-	300 °C	-	Self-producted NH_3_ atmosphere	2.78 eV ↓ 3.00 eV	6.57 m^2^/g ↓ 38.51 m^2^/g	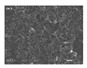	Photocatalytic degradation
[86]	Melamine 550 °C for 4 h 10 °C min^−1^	6 g	300 °C 2 °C min^−1^	1 h	Mass ratio of CN (B): KOH is 1:2 in 50 mL H_2_O	2.55 eV ↓ 2.66 eV	10.3 m^2^/g ↓ 265.2 m^2^/g	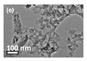	Visible-light-driven water splitting
[87]	Melamine 550 °C for 2 h in static air 5 °C min^−1^	0.1 g	350 °C 5 °C min^−1^	1.5 h in static air	0.1 g of bulk g-C_3_N_4_, 0.2 g of KOH and 5 mL of H_2_O	2.53 eV ↓ 2.75 eV	219 m^2^/g	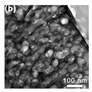	Photocatalytic hydrogen evolution

**Table 7:** Influencing factors, assistant methods, and results of post-hydrothermal preparation methods [70,75,76,90,91,92].

References	Synthesis of Bulk g-C_3_N_4_	Post-Hydrothermal				Assistant Method	Results			
		M_bulk g-C3N4_	Type of Solution	Treated Temperature	Treated Time		Band Gap	BET	Morphology	Application
[75]	Dicyandiamide 550 °C for 4 h 2.9 °C min^−1^	1.0 g	90 mL NaOH 0.12 M	120 °C	18 h	-	2.75 eV ↓ 2.67 eV	29.7 m^2^/g ↓ 64.7 m^2^/g	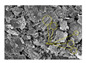	Photocatalytic oxidation of gaseous NO
[76]	Melamine 550 °C for 2 h 10 °C min^−1^	1.0 g	90 mL NaOH 0.1 M	130 °C	18 h	-	-	7.7 m^2^/g ↓ 53.7 m^2^/g	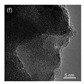	Photocatalytic NO oxidation in gas phase
[70]	Dicyandiamide 550 °C for 4 h 4 °C min^−1^ in air	0.2 g	(50-X) mL distilled water -ammonium hydroxides	120 °C	12 h	-	2.79 eV ↓ 2.91 eV	7.43 m^2^/g ↓ 42.78 m^2^/g	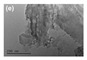	Photocatalytic hydrogen generation
[90]	Melamine 550 °C for 3 h 10 °C/min	0.5 g	35 mL ammonium hydroxide (mass fraction = 5%)	160 °C	4 h	-	2.76 eV ↓ 2.86 eV	14.6 m^2^/g ↓ 44.8 m^2^/g	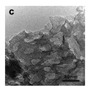	Photocatalytic hydrogen generation
[91]	Melamine 550 °C for 4 h 2.5 °C/min in N_2_	0.23 g	60 mL distilled water	180 °C	6 h	Porous g-C3N4 ---sealed condensation	2.68 eV ↓ 2.07 eV	1.59 m^2^/g ↓ 65.6 m^2^/g	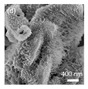	Overall water splitting
[92]	Polycondensation of urea	0.5 g	10 mL 0.1 M KOH	150 °C	12 h	Carbon thermal reduction	2.72 eV ↓ 2.57 eV	38.7 m^2^/g ↓ 197.0 m^2^/g	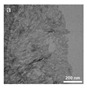	Photocatalytic hydrogen evolution

**Table 8:** Application of g-C_3_N_4_ systems for environmental pollutant mitigation [21,32,33,37,39,43,49,62,73,79,83,93,94,95,96,97].

References	Materials	Morphology	Synthesis	BET	Application
[21]	g-C_3_N_4_	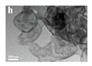	Pre-treatment	81.58 m^2^/g	H_2_ production activity and degradation rate
[32]	g-C_3_N_4_	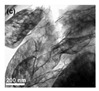	Pre-treatment	345 m^2^/g	Photocatalytic activity for NO removal
[33]	g-C_3_N_4_	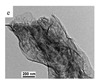	Pre-treatment	26.2 m^2^/g	Photocatalytic degradation
[37]	g-C_3_N_4_	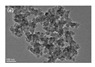	Pre-treatment	669.15 m^2^/g	Photocatalytic degradation
[43]	g-C_3_N_4_	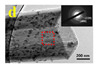	In-process	128 m^2^/g	Photocatalytic degradation
[39]	g-C_3_N_4_	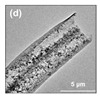	Pre-treatment	67.5 m^2^/g	Photocatalytic activity for NO removal
[49]	g-C_3_N_4_	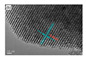	In-process	50.1 m^2^/g	Photocatalytic degradation of fluoroquinolone antibiotics
[62]	g-C_3_N_4_	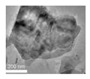	Post-treatment	25.7 m^2^/g	Photo-reduction of p-nitrophenol
[79]	g-C_3_N_4_	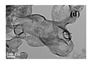	Post-treatment	151 m^2^/g	Visible light photocatalytic removal of NOx
[73]	g-C_3_N_4_	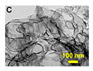	Post-treatment	142.8 m^2^/g	Photocatalytic degradation
[83]	g-C_3_N_4_	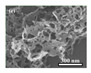	Post-treatment	114 m^2^/g	Photoeletrocatalytic Degradation of 4-Chlorophenol
[93]	g-C_3_N_4_	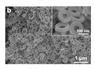	In-process	241.4 m^2^/g	Photocatalytic degradation
[94]	Mn@g-C_3_N_4_ /PANI/wood-derived carbon	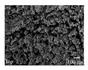	Composite materials	-	Photocatalytic degradation
[95]	g-C_3_N_4_	-	Pre-treatment	-	Photoelectrocatalytic degradation
[96]	g-C_3_N_4_@biogenic FeS	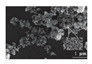	Composite materials	-	Photocatalytic degradation
[97]	NaYF_4_@g-C_3_N_4_	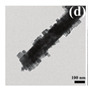	Composite materials	14.10 m^2^/g	Photocatalytic degradation

**Table 9:** The application of g-C_3_N_4_ nanostructures in energy conversion and storage [25,26,28,36,38,46,51,53,54,55,58,60,65,69,71,74,77,81,82,84,114,115,116,117,118,119].

References	Materials	Morphology	Synthesis	BET	Application
[28]	g-C_3_N_4_	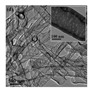	Pre-treatment	42.2 m^2^/g	Photocatalytic hydrogen evolution
[25]	g-C_3_N_4_	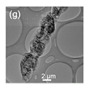	Pre-treatment	81.4 m^2^/g	Photocatalytic hydrogen evolution
[26]	g-C_3_N_4_	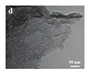	Pre-treatment	181.74 m^2^/g	Photocatalytic hydrogen evolution
[36]	g-C_3_N_4_	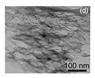	Pre-treatment	228.4 m^2^/g	Photocatalytic hydrogen evolution
[38]	g-C_3_N_4_	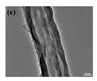	Pre-treatment	127.8 m^2^/g	Photocatalytic hydrogen evolution
[46]	g-C_3_N_4_	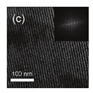	In-process	239 m^2^/g	Photocatalytic hydrogen evolution
[51]	g-C_3_N_4_	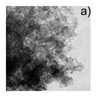	In-process	131 m^2^/g	Photocatalytic hydrogen evolution
[53]	g-C_3_N_4_	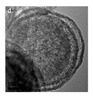	In-process	310.7 m^2^/g	Photocatalytic hydrogen evolution
[54]	g-C_3_N_4_	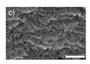	In-process	132.26 m^2^/g	Photocatalytic hydrogen evolution
[55]	g-C_3_N_4_	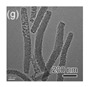	In-process	135.1 m^2^/g	Photocatalytic hydrogen evolution
[58]	g-C_3_N_4_	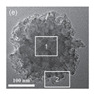	In-process	160 m^2^/g	Photocatalytic hydrogen evolution
[60]	g-C_3_N_4_	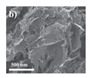	Post-treatment	55.4 m^2^/g	Photocatalytic hydrogen evolution and CO_2_ conversion
[65]	g-C_3_N_4_	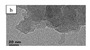	Post-treatment	205.8 m^2^/g	Photocatalytic hydrogen evolution
[71]	g-C_3_N_4_	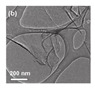	Post-treatment	384 m^2^/g	Hydrogen evolution
[69]	g-C_3_N_4_	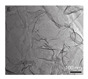	Post-treatment	306 m^2^/g	Photocatalytic hydrogen evolution
[74]	g-C_3_N_4_	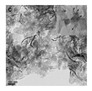	Post-treatment	117.27 m^2^/g	Photocatalytic hydrogen evolution
[81]	g-C_3_N_4_	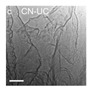	Post-treatment	99.73 m^2^/g	Photocatalytic hydrogen evolution
[77]	g-C_3_N_4_	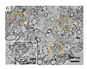	Post-treatment	277.98 m^2^/g	Photocatalytic hydrogen evolution
[82]	g-C_3_N_4_	-	Post-treatment	-	Lithium–sulfur batteries
[84]	g-C_3_N_4_	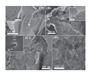	Post-treatment	196 m^2^/g	Photocatalytic hydrogen evolution
[114]	g-C_3_N_4_/Fe_2_TiO_5_	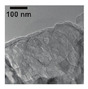	Composite materials	20.28 m^2^/g	CO_2_ Photoreduction
[115]	CN-Nv-C_3_N_4_	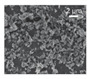	Post-treatment	-	Lithium metal batteries
[116]	g-C_3_N_4_ S-Scheme Homojunction	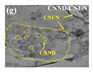	Post-treatment	122.04 m^2^/g	CO_2_ photoreduction
[117]	Pt NP decorated C_3_N_4_	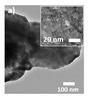	Composite materials	106.19 m^2^/g	Photocatalytic hydrogen evolution
[118]	CK-CNC	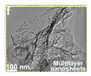	In-process	158.65 m^2^/g	Low-temperature sodium-ion batteries
[119]	Li-C_3_N_4_	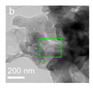	In-process	-	Oxygen reduction reactions
